# Small-molecule MMP2/MMP9 inhibitor SB-3CT modulates tumor immune surveillance by regulating PD-L1

**DOI:** 10.1186/s13073-020-00780-z

**Published:** 2020-09-28

**Authors:** Youqiong Ye, Xinwei Kuang, Zuozhong Xie, Long Liang, Zhao Zhang, Yongchang Zhang, Fangyu Ma, Qian Gao, Ruimin Chang, Heng-Huan Lee, Shuang Zhao, Juan Su, Hui Li, Jingbo Peng, Huifang Chen, Minzhu Yin, Cong Peng, Nong Yang, Jing Wang, Jing Liu, Hong Liu, Leng Han, Xiang Chen

**Affiliations:** 1 Department of Dermatology, Xiangya Hospital, Central South University, Changsha, 410008 Hunan China; 2 Shanghai Institute of Immunology, Department of Immunology and Microbiology, State Key Laboratory of Oncogenes and Related Genes, Shanghai Jiao Tong University School of Medicine, Shanghai, 200025 China; 3 Department of Biochemistry and Molecular Biology, The University of Texas Health Science Center at Houston McGovern Medical School, Houston, TX 77030 USA; 4 Hunan Key Laboratory of Skin Cancer and Psoriasis, Changsha, 410013 Hunan China; 5 Hunan Engineering Research Center of Skin Health and Disease, Changsha, 410008 Hunan China; 6 Xiangya Clinical Research Center for Cancer Immunotherapy, Central South University, Changsha, 410008 Hunan China; 7 Medical Genetics & School of Life Sciences, Central South University, Changsha, 410008 Hunan China; 8 Department of medical oncology, lung cancer and gastrointestinal unit, Hunan cancer hospital/The Affiliated Cancer Hospital of Xiangya School of Medicine, Central South University, Changsha, 410008 Hunan China; 9 Department of Health Management Center, Xiangya Hospital, Central South University, Changsha, 410008 Hunan China; 10 Department of Molecular and Cellular Oncology, The University of Texas MD Anderson Cancer Center, Houston, TX 77030 USA; 11 Department of Clinical Pharmacology, Xiangya Hospital, Central South University, Changsha, 410008 Hunan China; 12 Early Clinical Trial Center, Hunan Cancer Hospital and The Affiliated Cancer Hospital of Xiangya School of Medicine, Central South University, Changsha, 410008 Hunan China; 13 Research Center of Molecular Metabolomics, Xiangya Hospital, Central South University, Changsha, Hunan China

**Keywords:** Immune checkpoint blockade, Matrix metalloproteinases (MMPs), SB-3CT, Combination treatment

## Abstract

**Background:**

Immune checkpoint blockade (ICB) therapy has demonstrated considerable clinical benefit in several malignancies, but has shown favorable response in only a small proportion of cancer patients. Recent studies have shown that matrix metalloproteinases (MMPs) are highly associated with the microenvironment of tumors and immune cells. However, it is unknown whether MMPs are involved in immunotherapy.

**Methods:**

Here, we used integrative analysis to explore the expression landscape of the MMP family and its association with immune features across multiple cancer types. We used T cell cytotoxicity-mediated tumor killing assay to determine the co-cultured T cell activity of SB-3CT, an MMP2/9 inhibitor. We then used in vitro assays to examine the regulating roles of SB-3CT on PD-L1. We further characterized the efficacy of SB-3CT, in combination with anti-PD-1 and/or anti-CTLA4 treatment in mouse models with melanoma and lung cancer.

**Results:**

Our computational analysis demonstrated a strong association between MMP2/9 and immune features. We demonstrated that inhibition of MMP2/9 by SB-3CT significantly reduced the tumor burden and improved survival time by promoting anti-tumor immunity. Mechanistically, we showed that SB-3CT treatment significantly diminished both mRNA and protein levels of PD-L1 in cancer cells. Pre-clinically, SB-3CT treatment enhanced the therapeutic efficacy of PD-1 or CTLA-4 blockade in the treatment of both primary and metastatic tumors.

**Conclusions:**

Our study unraveled novel molecular mechanisms regarding the regulation of tumor PD-L1 and provided a novel combination therapeutic strategy of SB-3CT and ICB therapy to enhance the efficacy of immunotherapy.

## Background

Emerging immunotherapies that use immune checkpoint blockade (ICB) [1] have been approved to fight multiple cancers, including melanoma, non-small cell lung cancer, and bladder cancer. ICB therapies that target cytotoxic T lymphocyte–associated antigen 4 (CTLA-4) [2], programmed death 1 (PD-1) [3], and its ligand programmed death ligand 1 (PD-L1) [4] have shown promising clinical effects. However, only a small proportion of patients can benefit from anti-CTLA4 and/or anti-PD-1/PD-L1 immunotherapy. Recent studies have demonstrated the power of ICB in combination with other treatment strategies to improve treatment efficacy. For example, a combination of CPI-1205, which pharmacologically inhibits EZH2, plus ipilimumab increases the effectiveness of anti-CTLA-4 therapy in a mouse model [5]. In patients with unresectable stage III or IV melanoma, the combination of ipilimumab and sargramostim results in longer overall survival time and lower toxicity compared to treatment with ipilimumab alone [6]. Developing an efficient combinational approach necessitates a better understanding of the underlying mechanism for these synergistic effects. For example, epigenetic modifiers (e.g., BRD4, MLL1), noncoding RNAs (e.g., miR-200, miR570, LINK-A), signaling pathways (e.g., IFNγ-JAK-STAT, NF-κB, HIF-1α, EGFR, Hippo signaling pathway), and post-translational modification (GSK3β, CSN5) are involved in PD-1/PD-L1 pathways [7, 8].

Matrix metalloproteinases (MMPs) are a family of zinc-dependent endopeptidases [9]. There are 24 known MMPs in humans with several distinct domains, including gelatinases, collagenases, matrilysins, stromelysins, and membrane-type MMPs [10]. MMPs mediate a range of biological functions, such as degradation of various molecules for cell adhesion and modulation of cellular and extracellular matrix interactions [11]. Recent studies have shown that MMPs are highly associated with the microenvironment of tumors and immune cells and that targeting MMPs may overcome the barrier of immune suppression [11, 12]. For example, MMPs process CCL/MCP and CXCL chemokines and their receptors to modulate inflammatory and immune responses [13]. MMP9 is a component of the angiogenic switch during carcinogenesis [14], and MMP9-cleaved osteopontin fragments contribute to tumor immune escape by inducing the expansion of myeloid-derived suppressor cells (MDSCs) [15]. Clinical trials have pharmacologically targeted MMPs, and multiple broad-spectrum MMP inhibitors, such as batimastat, CGS 27023A, marimastat, prinomastat, tanomastat, and periostat [16], have been developed to target the synthesis, secretion, and activation of MMPs. Among these, marimastat, which was seen as the most promising, was evaluated in phase III trials for patients with breast cancer, lung cancer, and gastric cancer [17–19]. MMP inhibitors have failed to improve the overall survival times of patients or to alleviate symptomatic progression of cancer in clinical trials, mainly due to the non-specificity of the drug and the complex complicated background for specific effects of MMPs [20]. There is a need to understand the impact of MMPs in carcinogenesis in order to optimize the effects of MMP inhibitors in cancer therapy. In addition, the cross-talk between MMP inhibitors and ICB treatment remains unknown. In the present study, we systematically explored the genomic alterations of MMPs across 33 cancer types from The Cancer Genome Atlas (TCGA) [21]. We further characterized the efficacy of one MMP inhibitor, SB-3CT, in combination with anti-PD-1 and/or anti-CTLA4 in vitro and in vivo. Our research provides further understanding of MMPs involved in immunotherapy.

## Methods

### Data collection and processing

Normalized gene expression data based on the expectation maximization of 33 cancer types was downloaded from the TCGA data portal (http://gdac.broadinstitute.org/) [22]. Other independent expression datasets for patients with different cancer types were downloaded from Gene Expression Omnibus (https:// www.ncbi.nlm.nih.gov/geo/), including lung cancer (GSE33072 [23], GSE42127 [24]) and head and neck cancer (GSE65858 [25]). The gene expression data for liver cancer (LICA, LIRI) were downloaded from the International Cancer Genome Consortium (https://icgc.org/icgc/). The percentages of TILs were obtained from Thorsson et al. [26] (https://gdc.cancer.gov/about-data/publications/panimmune).

The gene list of MMPs was obtained from https://www.genenames.org/. MMP family members were divided into seven groups based on their typical structure [10]. The gene signature of the immune cell populations was obtained from Charoentong et al. [27]. Fifty hallmark gene sets were downloaded from The Molecular Signatures Database (MSigDB, http://software.broadinstitute.org/gsea/msigdb/) [28]. We used GSVA [29] to calculate the score of each MMP group, immune cell populations, and MSigDB hallmark pathways. MMP groups were considered to show differential expression between tumor and normal paired tissue samples if the GSVA score difference > 0.3 and the two-sided paired Student’s *t* test *p* < 0.05. We calculated Spearman’s correlation between the expression of immune features and the score of MMP groups, considering |*Rs*| > 0.2 and false discovery rate (FDR) < 0.05 for statistical significance.

The epithelial-to-mesenchymal transition gene signatures were obtained from Mak et al. [30], including 25 epithelial marker genes and 52 mesenchymal marker genes. The EMT score for each sample was estimated as ${\sum}_i^N{M}^i/N-{\sum}_j^n{E}^j/n$, as described in a previous study [30], where *M* and *E* represent the expression of the mesenchymal gene and epithelial gene, respectively, and *N* and *n* respectively represent the number of mesenchymal genes and epithelial genes.

### Cell culture and transfections

The human malignant melanoma cell lines (A375 and SK-MEL-28) and mouse LLC cell lines were cultured in Dulbecco’s modified Eagle’s medium supplemented with 10% fetal bovine serum (BI), 100 U of penicillin, and 100 μg/ml streptomycin (Gibco). The human A549 lung cancer and mouse melanoma cell line B16F10 was cultured in RPMI1640 medium. All cell lines were routinely tested for mycoplasma contamination and found to be negative. SB-3CT was added to the complete medium at the indicated concentrations and time.

### Plasmids and vectors

For the stable infection cell lines, MMP2 and MMP9 shRNA lentiviral vectors carrying hairpins were purchased from Shanghai GemmaPharma. The targeting sequences were described as follows:

shMMP2 #1:AAGAACCAGATCACATACAGG;

shMMP2 #2: AACGGACAAAGAGTTGGCAGT;

shMMP9 #1: AACATCACCTATTGGATCCAA;

shMMP9 #2: AGTACTGGCGATTCTCTGAGGC.

MMP2 and MMP9 overexpression plasmids were synthesized by Shanghai Genechem.

### Antibodies and chemicals

Human anti-PD-L1-Rb (ab213524) and mouse anti-PD-L1-Rb (ab213480) were purchased from Abcam; anti-CD8α-Rb (GB11068 and GB11068-1) was purchased from Servicebio; anti-PD-1-Rb (84651) was purchased from Cell Signaling Technology; the antibodies specific for human anti-MMP2-Rb (10375-2-AP), anti-MMP9-Rb (10373-2-AP), anti-MMP9-Rb (10373-2-AP), anti-GAPDH-M (60004-1-Ig), and anti-PD-L1-M (66248) were purchased from Proteintech; SB-3CT was purchased from Selleck(S7430); and in vivo mAb anti-PD-1-M (BE0146), anti-CTLA-4-M (BE0164), and IgG isotype control (BE0086, BE0089) were purchased from Bioxcell.

### T cell–mediated tumor cell killing assay

To acquire activated T cells, human peripheral blood mononuclear cells (LTS1077, Yanjin Biological) were cultured in CTSTM AIIM VTM SFM (A3021002; Gibco) with ImmunoCult Human CD3/CD28/CD2 T cell activator (10,970; STEMCELL Technologies) and IL-2 (1000 U/mL; PeproTech, Rocky Hill, NJ, USA) for 1 week according to the manufacturer’s protocol. The experiments were performed with anti-CD3 antibody (100 ng/mL; 16-0037; eBioscience, Thermo Scientific), IL-2 (1000 U/mL). Cancer cells were allowed to adhere to the plates overnight and then incubated for 48 h with activated T cells in the presence or absence of SB-3CT (25 μM). The ratios between cancer cells and activated cells (1:3) were modified according to the purpose of each experiment. T cells and cell debris were removed by phosphate-buffered saline (PBS) wash, and living cancer cells were then quantified by a spectrometer at OD (570 nm) followed by crystal violet staining.

### Flow cytometry analysis

In this study, all flow cytometry antibodies and agents were purchased from BioLegend (San Diego, CA, USA). In the mouse samples, single-cell suspension of B16f10-xenograft tumor was obtained by rapid and gentle stripping, physical grinding, and filter filtration. After blocking with CD16/CD32 (40477) antibody and removing dead cells with Zombie Red Fixable Viability Kit (77475), cells were stained using APCCY7-CD45(103116), AF700-CD3(100216), PECY5.5-CD4(100434), PECY7-CD8(100722), APC-CD25(102012), and BV421-PD1(135218) for 20 min. After fixation and permeabilization (421402), intracellular antibody was stained using PE-FOXp3(126404), FITC-GZMB (372206), and BV711-IFNγ(505836). To detect the expression of PD-L1 on the membrane of human cell lines, cells were stained using PE-PDL1 (329706), after blocking with Human TruStain FcX(422302) antibody and removing dead cells with Zombie Aqua Fixable Viability Kit (423102). Stained cells were analyzed by FACS Dxp AthenaTM (Cytek, Fremont, CA, USA). Data were further analyzed by Flow Jo 10.0 software.

### Immunoblot analyses

Cells were lysed in RIPA lysis buffer (DingGuo, China) supplemented with protease inhibitors and phosphatase inhibitors (Selleck, Houston, TX, USA). Protein concentrations were measured using a Beckman Coulter DU-800 spectrophotometer using the BCA reagent (Beyotime, China). Equal amounts of protein were resolved by SDS–PAGE and immunoblotted with indicated antibodies. The blots were detected using a gel image analysis system (LI-COR, Lincoln, NE, USA).

### Immunofluorescence

For multiple immunofluorescence, human tissue chip and 4-μm paraffin sections were baked for 120 min at 60 °C and then deparaffinized. Antigen was retrieved at EDTA antigen retrieval buffer (pH 8.0) and maintained at a sub-boiling temperature for 8 min, then left standing for 8 min and followed by maintaining at sub-boiling temperature again for 7 min. After spontaneous fluorescence quenching, samples were blocked in 3% BSA, PBS wash with 0.25% Triton X-100 for 1 h at room temperature. Primary antibodies targeting PD-1, CD8α, or PD-1 were incubated overnight at 4 °C in the blocking solution and then the following day were held at room temperature for 30 min. After extensive washing in PBS-0.25% Triton X-100, the secondary antibody was added to the blocking solution and incubated for 2 h if needed. Each primary antibody was dyed separately, and between intervals of each staining, the antigen was repaired once again in the EDTA antigen retrieval buffer (pH 8.0) by a microwave oven. After extensive washing in PBS-0.25% Triton X-100, slides were given a coverslip of anti-fade mounting medium. Then, the slides were incubated with DAPI solution at room temperature for 10 min and kept in a dark place. Microscopy detection was performed and images were collected by fluorescent microscopy.

### Real-time RT-PCR analyses

Total RNAs were extracted using trizol (Bioteke Corporation, China), and reverse transcription reactions were performed using HiScript II Q RT SuperMix for qPCR (Vazyme, China) according to the manufacturer’s instructions. Then, 40 cycles of quantitative reverse-transcription polymerase chain reaction (qRT-PCR) were conducted in 96-well plates using SYBR Green qPCR mixture (CWBIO, China) on the QuantStudio3 Real-Time PCR System. The fold change of gene expression was calculated by 2– (ΔCtexperimental group–ΔCtcontrol group). The sequence of primers was as follows:

Human GAPDH: forward, 5′-GGAGCGAGATCCCTCCAAAAT-3′; reverse, 5′-GGCTGTTGTCATACTTCTCATGG-3′.

Mouse GAPDH: forward, 5′-AGGTCGGTGTGAACGGATTTG-3′; reverse, 5′-GGGGTCGTTGATGGCAACA-3′.

Human PD-L1 (also known as CD274): forward, 5-TGGCATTTGCTGAACGCATTT-3′; reverse, 5′-TGCAGCCAGGTCTAATTGTTTT-3′.

Mouse PD-L1 (also known as Cd274): forward, 5′-GCTCCAAAGGACTTGTACGTG-3′; reverse, 5′-TGATCTGAAGGGCAGCATTTC-3′.

Human MMP9: forward, 5′-TGTACCGCTATGGTTACACTCG-3′; reverse, 5′-GGCAGGGACAGTTGCTTCT-3′.

Human MMP2: forward, 5′-TACAGGATCATTGGCTACACACC-3′; reverse, 5′-GGTCACATCGCTCCAGACT-3′.

### Mouse tumor generation and implantation

The animal protocol was approved by the Ethics Committee of Xiangya Hospital (Central South University, Changsha, Hunan, China). All experiments strictly followed the guidelines for the investigation of experimental pain in conscious animals for minimizing animals’ suffering and improving animals’ welfare. Wild-type B16F10 (5 × 10^5^) or LLC cells (1 × 10^6^) were injected subcutaneously into specific-pathogen-free-grade 6-week-old C57BL/6 female mice (from the Shanghai SLAC). Nearly 1 week later, the mice were pooled and randomly divided into several groups. When establishing the lung metastatic xenograft model, the mice in each group received 100-μl cell suspension (including 5 × 10^5^ B16F10 cells) via intravenous injection and were administered treatment on day 3. Mice were treated daily with SB-3CT (10 mg/kg, i.p.), anti-mouse PD-1 mAb (100 μg/mouse/3 days) or anti-mouse CTLA-4 mAb (200 μg/mouse/3 days), combination therapy or control vehicle/isotype only, for 9–15 days. Subsequently, tumors were collected and analyzed by FACS. The excised xenografts were also snap-frozen in liquid nitrogen. Paraffin-embedded tumor blocks were prepared for further analysis at the same time.

### RNA-seq analysis

RNA-seq was performed by Illumina Hiseq x-ten with 150-base paired-end reads. All reads were aligned to the mouse reference genome (mm10 or hg19) using hisat2 [31] with the default setting. Stringtie [32] was used to calculate the transcriptional expression level as fragments per kilobase per million. Genes were considered to be differentially expressed if the |log_2_(fold change)| > 0.5 and two-sided Student’s *t* test *p* < 0.5.

### Survival analysis

Overall survival analyses were performed using the R package survival, and the subjects were dichotomized based on median expression (enrichment score) or divided in two or more groups by specified parameters. Kaplan–Meier estimation of survival was used to construct the survival curves. Log-rank tests (corresponding to a two-sided *Z* test) were used to compare overall survival between subjects in different groups, and the hazard ratio (HR) (95% confidence interval) was provided for comparison of two groups. The *p* values were adjusted for multiple testing based on the FDR according to the Benjamini–Hochberg method.

### Statistical analysis

Experimental data were expressed as mean ± s.d. One-way ANOVA and Dunnett’s multiple comparison test were used to determine the statistical differences between multiple groups, and two-sided Student’s *t* test was used in two groups. A *p* value of less than 0.05 was considered statistically significant, with the analysis and mapping by Graphpad Prism software (GraphPad Software, Inc., version 7.0).

## Results

### Dysregulation of MMP family and association with cancer hallmarks

**Fig. 1:**
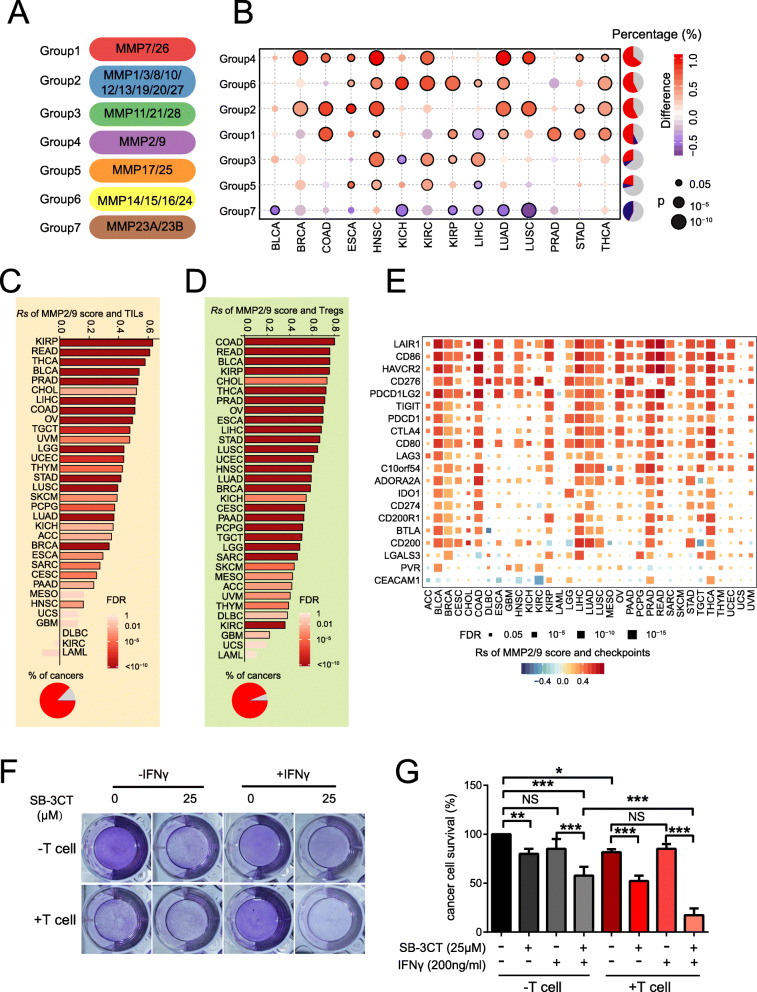
Dysregulation of MMPs and associations with cancer hallmarks. **a** Seven MMP groups based on their typical structures. **b** Differential score of MMP groups across 14 cancer types (*Y*-axis) compared to paired normal samples (fold change > 1.5; paired two-sided Student’s *t* test *p* < 0.05). Pie chart in the right panel shows the percentage of cancer types with significant upregulation (red), downregulation (blue), and non-significant alteration (gray). **c**, **d** MMP2/9 score correlated with **c** T cell–tumor infiltrating lymphocytes (TILs) and **d** regulatory T cells (Tregs). Pie charts show the percentage of cancer types with positive (red) and non-significant (gray) correlation. **e** Spearman’s correlation of MMP groups and inhibitory immune checkpoints. **f**, **g** T cell–mediated cancer cell killing assay. **f** SK-MEL-28 melanoma cells co-cultured with activated T cells for 48 h with or without SB-3CT (12.5, 25 μM) or IFNγ (200 ng/mL) were subjected to crystal violet staining. SK-MEL-28-to-T cell ratio, 1:3. **g** Statistical analysis. Results are mean ± s.d. NS, *p* > 0.05, *p < 0.05, **p < 0.01, and ***p < 0.001, as determined by one-way ANOVA and Dunnett’s multiple comparison test

Dysregulation of MMPs contributes to cancer development [33]. The MMP family can be divided into seven groups based on their typical structure [10]: group1 (MMP7/26), group2 (MMP1/3/8/10/12/23/19/20/27), group3 (MMP11/21/28), group4 (MMP2/9), group5 (MMP17/25), group6 (MMP14/15/16/24), and group7 (MMP23A/23B) (Fig. 1a). We calculated the expression levels of each group by gene set variation analysis (GSVA) score [29] and compared between paired tumor and normal tissue samples across 14 cancer types from TCGA [22]. We observed that each MMP family group is differentially expressed in at least one cancer type (Fig. 1b), which suggests frequent dysregulation of MMPs in cancer. Six of the 7 MMP groups tended to be overexpressed in multiple cancer types. Among these, group4 (MMP2 and MMP9) was upregulated in nine cancer types, including breast invasive carcinoma (BRCA; diff = 0.86; *p* = 1.6 × 10^−14^), lung adenocarcinoma (LUAD; diff = 1.0; *p* = 3.32 × 10^−12^), lung squamous cell carcinoma (LUSC; diff = 0.87; *p* = 1.8 × 10^−7^), and head and neck squamous cell carcinoma (HNSC; diff = 0.97; *p* = 5.2 × 10^−15^). We further performed survival analyses and demonstrated that high scores for group4 are associated with worse survival in bladder urothelial carcinoma (BLCA; *p* = 0.37), kidney renal clear cell carcinoma (KIRC; *p* = 0.046), low-grade glioma (LGG; *p* = 1.1 × 10^−4^), and uveal melanoma (UVM; *p* = 0.012). In addition, mRNA expression of MMP2 and MMP9 is associated with worse survival in multiple cancer types (Additional file 1: Fig. S1A and B).

To determine the functional effects of group4 dysregulation in human cancer, we analyzed the correlation between the group4 GSVA score and 50 cancer hallmark gene sets, which represent well-defined biological processes in human cancers [28]. We found that the group4 score highly correlated with multiple cancer hallmarks, including angiogenesis, apoptosis, epithelial–mesenchymal transition (EMT), and inflammatory response (Additional file 1: Fig. S1C). Interestingly, this score significantly correlated with many cancer hallmarks associated with immune response, including TNF-α signaling via NF-κB, IL2, and STAT5 signaling; IL6, JAK, and STAT3 signaling; inflammatory responses; IFNα response; and IFNγ response (Additional file 1: Fig. S1C). Therefore, we further explored the association of MMP2/9 with the tumor–immune environment. We observed that MMP2/9 highly correlated with tumor infiltration lymphocytes (TILs) across 27 cancer types (median Spearman’s correlation coefficient [*Rs*] = 0.43; Fig. 1c). Furthermore, MMP2/9 correlated with regulatory T cells (Tregs) [34, 35] across 30 cancer types (median *Rs* = 0.57; Fig. 1d). In addition, MMP2/9 positively correlated with most inhibitory immune checkpoints across multiple cancer types (Fig. 1e). For example, MMP2/9 highly correlated with exhausted T cell marker PDCD1 across 23 cancer types. These results suggested that MMP2/9 may contribute to tumor immune escape.

To further investigate the functional role of MMP2/9 in the immune response, we performed an in vitro T cell cytotoxicity-mediated tumor killing assay based on SK-MEL-28 melanoma cells in the presence of increasing concentrations of SB-3CT, which is known to inhibit gelatinases (MMP-2 and MMP-9) with high selectivity [36, 37]. Inhibition of MMP2/9 significantly increased the CD8^+^ T cell cytotoxicity and ability to eliminate tumor cells (Fig. 1f, g), and the CD8^+^ T cell cytotoxicity and killing ability was significantly stronger with IFNγ induction, which induced PD-L1 surface expression [38]. Taken together, our T cell tumor killing assay suggested that SB-3CT, an MMP2/9 inhibitor, can activate co-cultured T cell activity.

### SB-3CT, the MMP2/9 inhibitor, enhances the therapeutic efficacy of PD-1 blockade

**Fig. 2:**
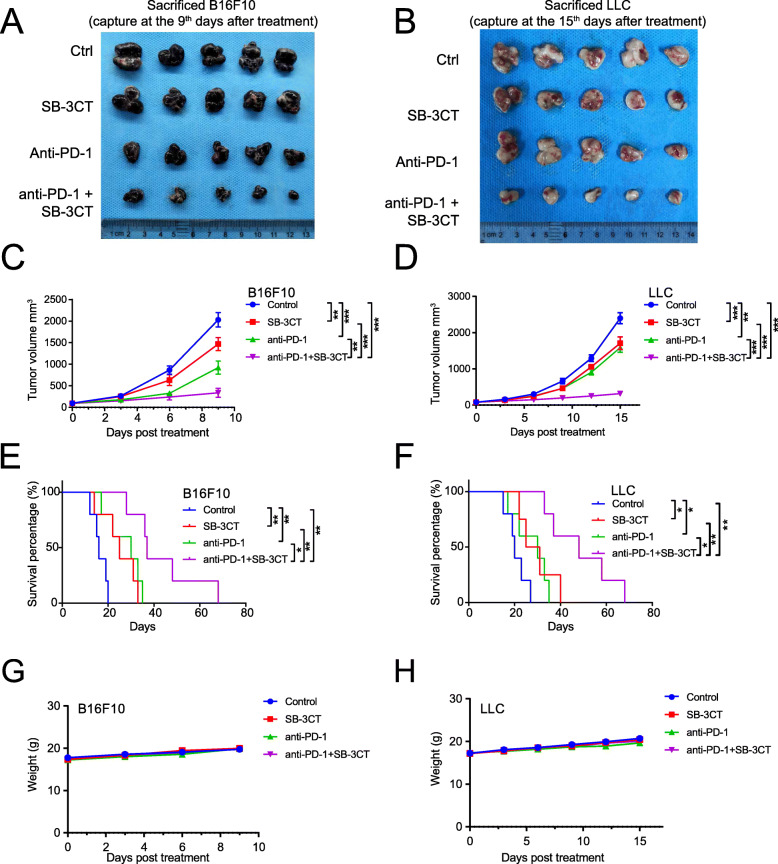
Synergistic therapeutic effect of combination treatments with MMP2/9 inhibitor, SB-3CT, and anti-PD-1. Images of **a** B16F10 tumor and **b** LLC tumor, collected from the combination strategies (control: isotype; PD1 inhibition: anti-PD1; MMP2/9 inhibition: SB-3CT; combination treatment: anti-PD-1+SB-3CT). The B16F10 tumor was captured at the ninth days, and the LLC tumor was captured at the fifth days after treatment. Tumor volumes of **c** B16F10 and **d** LLC tumor-bearing C57/BL6 mice treated with isotype, SB-3CT, anti-PD-1, or combination strategy. Kaplan–Meier survival analysis of **e** B16F10 or **f** LLC tumor-bearing C57/BL6 mice treated with isotype, SB-3CT, anti-PD-1, or combination strategy. Body weight of **g** B16F10 and **h** LLC tumor-bearing C57/BL6 mice treated with isotype, SB-3CT, anti-PD-1, or combination strategy. Sample size per group in one experiment is 5. Error bars represent s.d. of individual mice per group in one experiment. NS, *p* > 0.05, *p < 0.05, **p < 0.01, and ***p < 0.001, as determined by **c**, **d** two-way ANOVA, **e**, **f** two-sided log-rank test, and **g**, **h** one-way ANOVA

With the in vitro evidence that SB-3CT, the MMP2/9 inhibitor, increased CD8^+^ T cell cytotoxicity, we further investigated whether the effect of SB-3CT was synergistic with that of anti-PD-1 therapy in vivo. We applied SB-3CT in combination with anti-PD-1 antibody in tumor mouse models of B16F10 melanoma and Lewis lung carcinoma (LLC; Additional file 1: Fig. S2). In brief, mice were injected subcutaneously with B16F10 tumor cells (5 × 10^5^) or LLC tumor cells (1 × 10^6^). Afterwards, mice with tumors reaching about 100 mm^3^ in size were randomized into four groups with different treatment strategies: control (isotype), treatment with anti-PD-1 antibody, treatment with SB-3CT, and the combination treatment of anti-PD-1 and SB-3CT. Treatments with anti-PD-1 were administered every 3 days and treatments with SB-3CT were administered every day until tumor capture at the ninth days, and tumor growth was measured every 3 days. In the B16F10 melanoma model, SB-3CT alone significantly decreased tumor growth at day 9 after treatment (mean tumor size 1469 vs 2030 mm^3^; *p* < 0.01 by two-way analysis of variance (ANOVA), Tukey’s multiple comparison test; Fig. 2a, c), while a combination treatment of SB-3CT and anti-PD-1 achieved better efficacy (mean tumor size 339 vs 2030 mm^3^; *p* < 0.001; Fig. 2a, c). SB-3CT alone substantially extended the overall survival time (median survival 31 vs 19 days; *p* < 0.01, log-rank test) of B16F10 tumor-bearing mice, and combined with anti-PD-1, enhanced the survival benefit (median survival 48 vs 19 days; *p* < 0.001; Fig. 2e). The LLC model showed similar results. Mice given the SB-3CT treatment had inhibited tumor growth (1709 vs 2397 mm^3^; *p* < 0.001; Fig. 2b, d) and extended survival time (25 vs 16 days; *p* < 0.05; Fig. 2f), while the combination treatment enhanced the decrease in tumor growth (316 vs 2397 mm^3^; *p* < 0.001) and extended survival (37 vs 16 days; *p* < 0.01; Fig. 2f). More importantly, administration of SB-3CT alone or in combination did not result in a significant change in body weight (Fig. 2g, h), which suggested that SB-3CT treatments in tumor-bearing mice had limited toxicity. Taken together, our in vitro study showed that SB-3CT, the MMP2/9 inhibitor, enhanced the therapeutic efficacy of PD-1 blockade.

### Enhanced activation of lymphocytes and reduced expression of PD-L1 with SB-3CT treatment

**Fig. 3:**
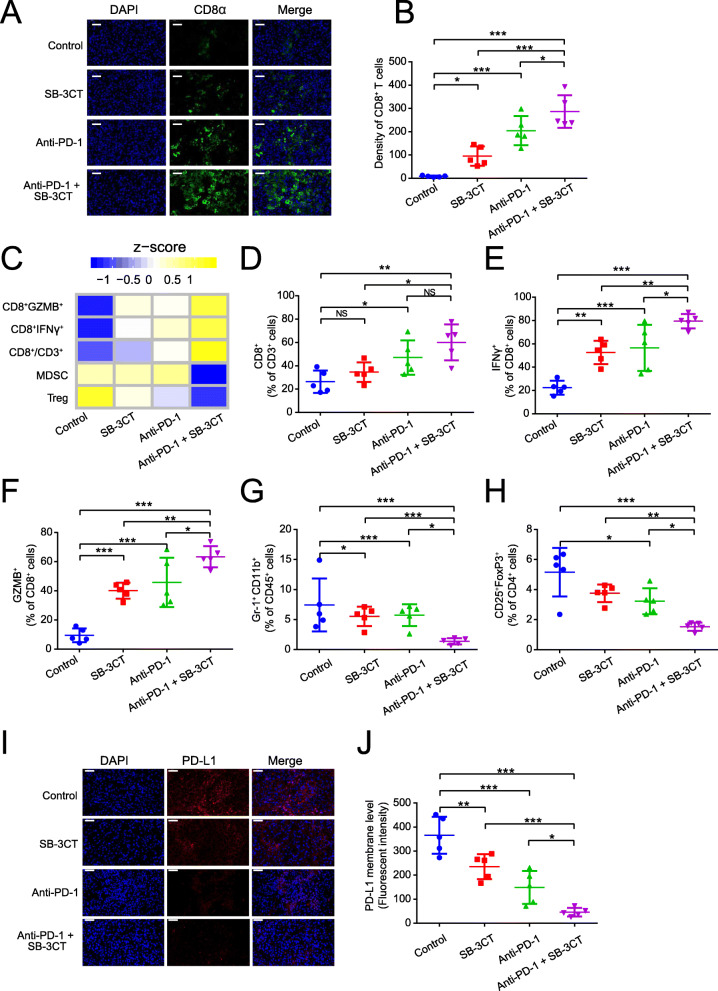
Immune features in tumors for B16F10 xenograft mouse model with SB-3CT treatment. **a** Fluorescence expression and **b** quantification of CD8^+^ T cells. **c** Heatmap of *Z*-score normalized percentage of immune cell populations (**d**–**h**) in TILs for B16F10 tumor-bearing mice treated with anti-PD-1 and SB-3CT in combination or alone. **d**–**h** In the implanted B16F10 tumors from mice treated with or without SB-3CT and PD-1 blockade, fluorescence-activated cell sorting (FACS) was used to measure **d** CD8^+^ in CD3^+^ T cells, **e** CD8^+^ IFNγ^+^ in CD8^+^ T cells, **f** CD8^+^ GZMB^+^ in CD8^+^ T cells, **g** Gr-1^+^ CD11b^+^ MDSCs in CD45^+^ cells, and **h** CD25^+^ FOXP3^+^ Treg in CD4^+^ cells. **i** Fluorescence expression and **j** quantification of PD-L1 in B16F10 tumor-bearing wild-type C57/BL6 mice treated with isotype, SB-3CT, anti-PD-1, or combination strategy. Sample size is 5 in each cohort. Scale bars, 50 μm. Results are mean ± s.d. ns, *p* > 0.05,*p < 0.05, **p < 0.01, and ***p < 0.001, as determined by one-way ANOVA and Dunnett’s multiple comparison test

To elucidate the alteration of immune features in the enhanced therapeutic efficacy of SB-3CT, we investigated the tumor–immune microenvironment of B16F10 tumor-bearing mice treated with anti-PD-1 and SB-3CT in combination or alone. Immunofluorescent staining showed that the combination treatment substantially increased the population of activated tumor infiltrating CD8^+^ T cells in the tumor (Fig. 3a, b). Furthermore, fluorescence-activated cell sorting (FACS) analysis demonstrated the status of immune infiltration in tumors after treatment (Figs. 3c–h; Additional file 1: Fig. S3). The combination treatment significantly increased the infiltration percentage of CD8^+^ T cells (26.3 to 52.4% of CD3^+^ cells; *p* < 0.01 by one-way ANOVA, Tukey’s multiple comparison test; Fig. 3d). We further assessed the functional consequence of SB-3CT and anti-PD-1 combination in TILs. Compared to a control group, SB-3CT treatment significantly improved the IFNγ^+^/CD8^+^ T cell infiltration (from 23.3 to 54.7%; *p* < 0.01), which was synergistically improved by combination treatment of SB-3CT and anti-PD-1 (23.3 to 80.0%; *p* < 0.001; Fig. 3e). The cytolytic capacity of CD8^+^ TILs was also determined by granzyme B (GZMB) expression. GZMB^+^/CD8^+^ T cell infiltration was significantly improved with SB-3CT treatment (from 7.43 to 40.9%; *p* < 0.001) and synergistically improved by the combination treatment (from 7.43 to 64.0%, *p* < 0.001; Fig. 3f). Meanwhile, the combination treatment substantially reduced the infiltration of suppressed immune cell populations. For example, the percentage of Gr-1^+^ CD11b^+^ MDSCs in CD45^+^ cells was reduced from 5.95 to 1.75% (*p* < 0.001; Fig. 3g), and the percentage of CD25^+^FOXP3^+^Treg cells in CD4^+^ TILs was reduced from 6.35 to 1.74% (*p* < 0.001; Fig. 3h). Furthermore, immunofluorescent staining showed that PD-L1 protein expression was significantly reduced upon SB-3CT treatment, and the combination treatment further reduced the protein expression of PD-L1 (Fig. 3i, j). Taken together, these results suggested that SB-3CT and anti-PD-1 treatment substantially improved the immune cell infiltration and cytotoxicity of T cells and reduced the infiltration of suppressed immune cells. Our results suggested a reduction of PD-L1 expression upon SB-3CT treatment.

### SB-3CT modulates PD-L1-related pathways through diminishing PD-L1

**Fig. 4:**
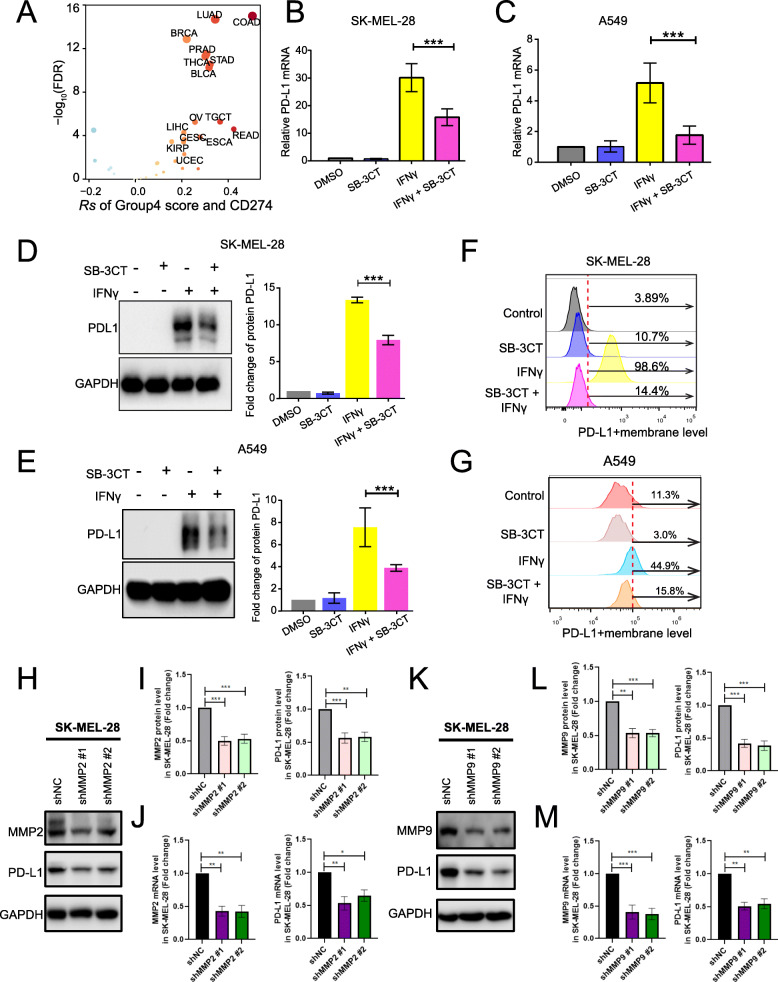
Downregulation of MMP2/9 by SB-3CT treatment reduced PD-L1 expression. **a** Spearman’s correlation of group4 score and PD-L1 mRNA expression across 33 cancer types. **b**–**g** Evaluation of PD-L1 expression derived from SK-MEL-28 melanoma cell lines and A549 lung cancer cell lines treated with DMSO, SB-3CT (25 μM), IFNγ (200 ng/mL), and IFNγ/SB-3CT in combination for 24 h. **b**, **c** PD-L1 expression by RT-PCR in **b** SK-MEL-28 melanoma cell lines and **c** A549 lung cancer cell lines. **d**, **e** Western blot (left panel: representative images, right panel: quantification) of PD-L1 protein levels in **d** SK-MEL-28 and **e** A549. **f**, **g** Flow cytometry of PD-L1^+^ membrane level in **f** SK-MEL-28 and **g** A549. **h**–**m** PD-L1 expression of SK-MEL-28 melanoma cell line transfected with shMMP2 (**h**–**j**) and shMMP9 (**k**–**m**) or the scrambled negative control shRNA (shNC). Western blot (**h**, **k**) quantification of MMP2, MMP9, and PD-L1 protein expression (**i**, **l**), and RT-PCR analysis of MMP2, MMP9, and PD-L1 mRNA expression (**j**, **m**)

We observed decreased expression of PD-L1 upon treatment with SB-3CT in vivo. We hypothesized that MMP2/9, the target of SB-3CT, is a potential regulator of PD-L1. Indeed, the group4 (MMP2/9) score significantly correlated with the mRNA expression of PD-L1 across 33 TCGA cancer types (Fig. 4a), including LUAD (*Rs* = 0.37, *p* = 1.01 × 10^−15^). We observed similar positive correlation in multiple independent datasets, including lung cancer (*Rs* = 0.30, *p* = 5.9 × 10^−4^), head and neck cancer (*Rs* = 0.30, *p* = 4.1 × 10^−7^), and liver cancer (*Rs* = 0.40, *p* = 4.0 × 10^−10^; Additional file 1: Fig. S4A). Furthermore, SB-3CT led to a decrease in IFNγ-induced PD-L1 surface expression in two melanoma cell lines, SK-MEL-28 (Fig. 4b) and A375 (Additional file 1: Fig. S4B), and one lung cancer cell line, A549 (Fig. 4c). Using western blot, we further confirmed that SB-3CT treatment led to the downregulation of IFNγ-induced PD-L1 in total cellular protein levels in these cells (Fig. 4d, e and S4C). Flow cytometry analysis also showed downregulation of IFNγ-induced PD-L1 surface expression in SK-MEL-28 (from 98.6 to 14.4%; Fig. 4f), A375 (from 97.6 to 65.2%; Additional file 1: Fig. S4D), and A549 cells (from 44.9 to 15.8%; Fig. 4g). Collectively, these data demonstrated that SB-3CT treatment reduced IFNγ-inducible PD-L1 expression in a variety of cancer cell lines.

**Fig. 5:**
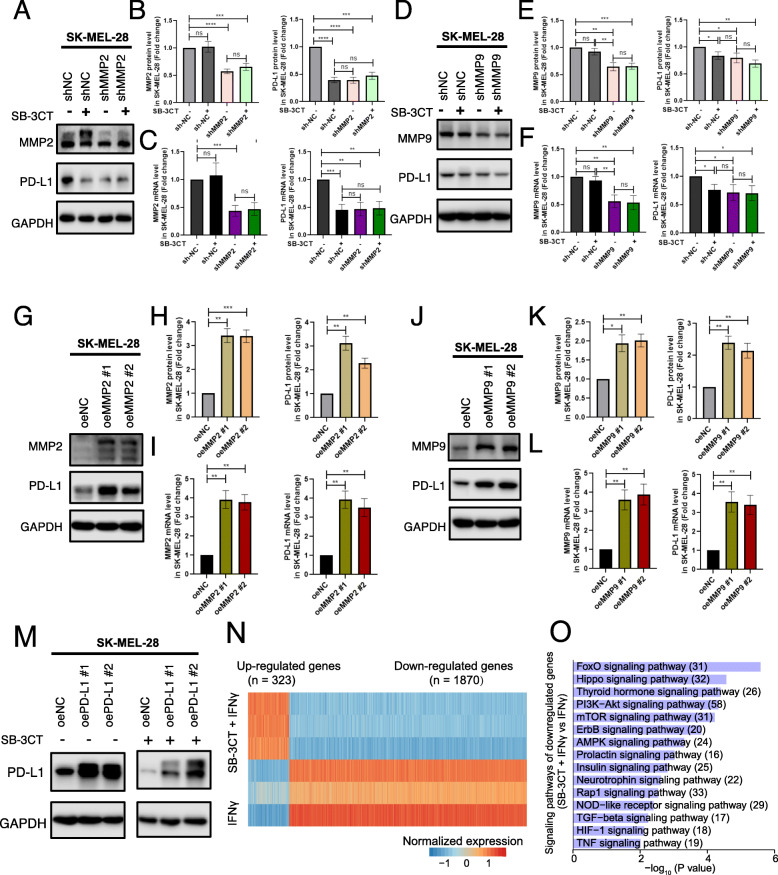
Regulation of PD-L1 expression through MMP2/9 has an anti-tumor effect. **a**–**f** Analysis of PD-L1 expression of SK-MEL-28 melanoma cell line with overexpression (oe) MMP2 (**a**–**c**), oeMMP9 (**d**–**f**). Western blot (**a**, **d**), quantification (**b**, **e**), and RT-PCR analysis (**c**, **f**) of MMP2, MMP9, and PD-L1 protein or mRNA expression. **g**–**l** PD-L1 expression of shMMP2 (**g**–**i**) and shMMP9 (**j**–**l**) SK-MEL-28 melanoma cell line treated with SB-3CT. Western blot (**g**, **j**); quantification of MMP2, MMP9, and PD-L1 protein (**h**, **k**); and mRNA expression (**i**, **l**). **m** Western blot showed the protein expression of PD-L1 for SK-MEL-28 melanoma cells with overexpression of PD-L1, treated with or without SB-3CT. **n** *Z*-scale normalization expression of differentially expressed genes (fold change > 1.5 and two-sided Student’s *t* test *p* < 0.05) between A375 melanoma cell lines treated with IFNγ/SB-3CT in combination and IFNγ. **o** Enriched signaling pathways for genes downregulated in A375 melanoma cell lines treated with SB-3CT and IFNγ in combination (Fisher’s exact test *p* < 0.05). All experiments were repeated three times independently. Results are mean ± s.d. NS, *p* > 0.05, *p < 0.05, **p < 0.01, and ***p < 0.001, as determined by one-way ANOVA and Dunnett’s multiple comparison test

To determine the downregulation of PD-L1 expression under SB-3CT treatment through the MMP2/9 inhibition, we knocked down (KD) MMP2 or MMP9 with two shRNAs in SK-MEL-28 (Fig. 4h–m) and A375 (Additional file 1: Fig. S4E-J) melanoma cell lines, respectively. We observed that two melanoma cell lines transfected with shMMP2 significantly decreased the PD-L1 mRNA and protein levels compared to cell lines transfected with the scrambled negative control (shNC) group (Fig. 4h–j; Additional file 1: Fig. S4E-G). Additionally, SK-MEL-28 and A375 melanoma cell lines with MMP9 KD also significantly decreased the PD-L1 mRNA and protein levels (Fig. 4k–m; Additional file 1: Fig. S4D-F). Furthermore, to test whether SB-3CT have any activity in the context of KD of MMP2 or MMP9, we treated melanoma cell lines (SK-MEL-28 and A375) transfected with shMMP2, shMMP9, or shNC with SB-3CT. The result showed SB-3CT treatment induced the reduction of PD-L1 levels in shNC cells. However, SB-3CT treatment could not further decrease PD-L1 levels in MMP2 or MMP9 KD cell lines (Fig. 5a–f; Additional file 1: Fig. S5A-F), indicating that SB-3CT have limited activity for PD-L1 expression in the context of KD of MMP2 and MMP9. In addition, to validate the regulation of PD-L1 expression through MMP2/9, we generated the stable overexpression (OE) MMP2 or MMP9 melanoma cell lines. We found that two melanoma cell lines with oeMMP2 or oeMMP9 significantly increased the PD-L1 mRNA and protein levels (Fig. 5g–l; Additional file 1: Fig. S5G-L). We further generated the PD-L1 overexpression (OE) cell lines in SK-MEL-28 and treated cells with or without SB-3CT. We observed that SB-3CT treatment significantly reduced the PD-L1 protein level in PD-L1 overexpression cell line (Fig. 5m). Our results suggest that SB-3CT indeed selectively inhibit MMP2/9 and result in the decreased expression of PD-L1.

To further understand the regulation of the MMP2/9 inhibitor, SB-3CT, on PD-L1 expression, we performed RNA-seq to identify the signaling pathways altered by SB-3CT in A375 cells. We found 323 significantly upregulated genes and 1870 downregulated genes upon treatment with SB-3CT (Fig. 5n). In particular, the downregulated genes were significantly enriched in oncogenic pathways, including PI3K-Akt, AMPK, Hippo, HIF-1, and mTOR signaling pathways (Fig. 5o), which have been reported as activating the expression of PD-L1 [7]. Taken together, our results suggested a potential mechanism in that SB-3CT decreases multiple oncogenic pathways, including the PI3K-AKT pathway, to decrease the expression level of PD-L1.

### Combination of SB-3CT and anti-CTLA-4 improves anti-tumor immune response

**Fig. 6:**
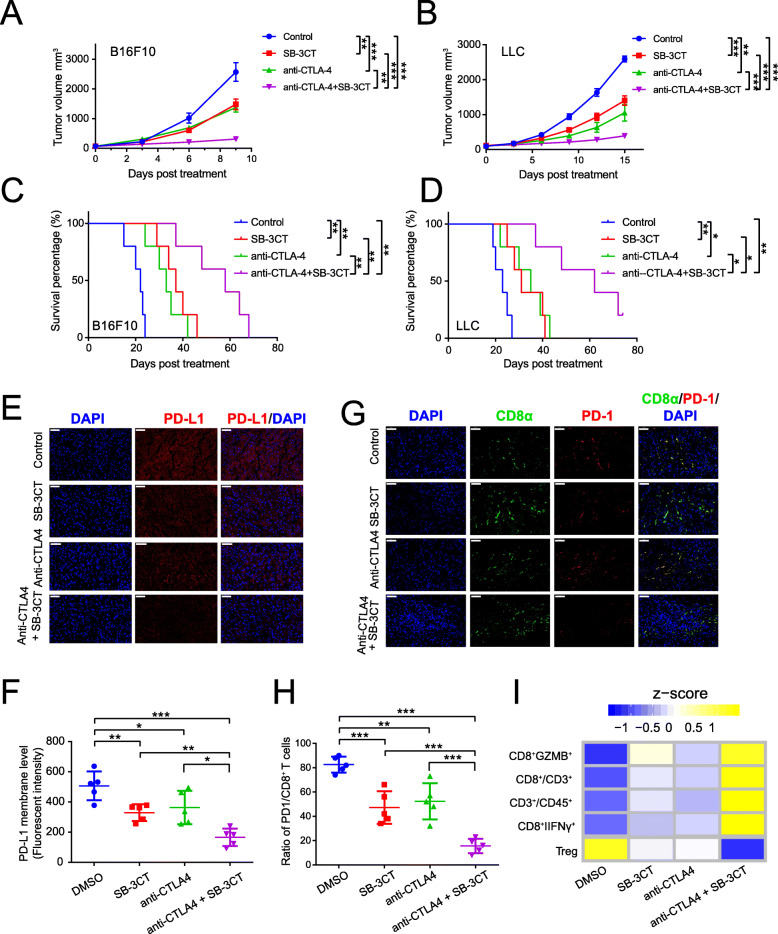
Synergistic therapeutic effect of SB-3CT treatment combined with CTLA-4 blockade. Tumor volumes of **a** B16F10 or **b** LLC tumor-bearing C57/BL6 mice treated with isotype, SB-3CT, anti-CTLA-4, or combination strategy. Kaplan–Meier survival analysis of **c** B16F10 or **d** LLC tumor-bearing C57/BL6 mice treated with isotype, SB-3CT, anti-CTLA-4, or combination strategy. **e**–**i** Immune features in B16F10 tumor-bearing C57/BL6 mice treated with isotype, SB-3CT, anti-CTLA-4, or combination strategy. **e** Fluorescence expression and **f** quantification of PD-L1 expression in tumor. **g** Fluorescence expression and **h** quantification of PD1^+^CD8^+^ T cells in tumor. **i** Heatmap represents *Z*-score normalized percentage of immune cell populations. Sample size per group in one experiment is 5. Error bars represent s.d. of individual mice per group in one experiment. NS, *p* > 0.05, *p < 0.05, **p < 0.01, and ***p < 0.001, as determined by **a**, **b** two-way ANOVA, **c**, **d** two-sided log-rank test, and **f**, **h** one-way ANOVA and Dunnett’s multiple comparison test

A recent study demonstrated the therapeutic efficacy of anti-PD-1 in combination with anti-CTLA4 [39]. Since we identified that SB-3CT can reduce PD-L1 expression, we hypothesized that the combination of SB-3CT and anti-CTLA-4 will also achieve therapeutic efficacy. We assessed the therapeutic efficacy of the MMP2/9 inhibitor SB-3CT in combination with the anti-CTLA-4 antibody in two tumor models, B16F10 melanoma (Fig. 6a, Additional file 1: Fig. S6A) and LLC (Fig. 6b, Additional file 1: Fig. S6B). In the B16F10 melanoma model, SB-3CT alone significantly decreased tumor growth at day 9 post-treatment compared to the control group (mean tumor size 1482 vs 2570 mm^3^; *p* < 0.01), while a combination treatment of SB-3CT and anti-CTLA-4 reached better efficacy (mean tumor size310 vs 2570 mm^3^; *p* < 0.001; Fig. 6a and Additional file 1: Fig. S6C). SB-3CT alone substantially extended the overall survival time (median survival37 vs 22 days; *p* < 0.01) of B16F10 tumor-bearing mice, and combined with anti-CTLA-4, enhanced the survival benefit (median survival time: 58 vs 22 days; *p* < 0.001; Fig. 6c). The LLC model showed similar results in that mice given the SB-3CT treatment had inhibited tumor growth (1402 vs 2593 mm^3^; *p* < 0.001; Fig. 6b and Additional file 1: Fig. S6D) and extended survival time (31 vs 23 days; *p* < 0.01; Fig. 6d), while the combination treatment enhanced the decrease in tumor growth (392 vs 2593 mm^3^; *p* < 0.001; Fig. 6b and Additional file 1: Fig. S6D) and extended survival (62 vs 23 days; *p* < 0.01; Fig. 6d). Administration of SB-3CT or in combination with CTLA-4 blockade exhibited minimal effects on the body weight of the mice (Additional file 1: Fig. S6E-F), which further confirmed the limited toxicity associated with SB-3CT treatment in tumor-bearing mice.

We further delineated the effects of SB-3CT and anti-CTLA-4 treatment in combination or alone on the tumor–immune microenvironment. Immunofluorescent staining showed that the PD-L1 protein was significantly reduced in SB-3CT-treated mice compared with the control group, and combination therapy enhanced this decrease (Fig. 6e, f). The combination treatment substantially reduced PD-L1 expression and increased the activated tumor infiltrating CD8^+^ T cell population in the tumor (Fig. 6g, h). Further FACS analysis revealed the functional activity of CD8^+^ TILs in tumors treated with the combination treatment (Fig. 6i, Additional file 1: Fig. S7, and Fig. S8). The combination treatment significantly increased the infiltration percentage of CD3^+^ T cells in CD45^+^ cells (from 23.6 to 52.0%; *p* < 0.01)(Additional file 1: Fig. S7A and C) and the infiltration percentage of CD8^+^ T cells in CD3^+^ T cells (from 40.4 to 69.7%; *p* < 0.001) (Additional file 1: Fig. S7B and D). We further assessed the functional consequences of SB-3CT and anti-CTLA-4 in combination in TILs and found that the combination treatment significantly enhanced the infiltration of IFNγ^+^CD8^+^ T cells (from 27.1 to 51.8%; *p* < 0.001; Additional file 1: Fig. S8A and D) and IFNγ^+^CD8^+^ T cells (from 7.43 to 64.0%, *p* < 0.001; Additional file 1: Fig. S8B and E). Meanwhile, the combination treatment substantially reduced the infiltration percentage of CD25^+^FOXP3^+^Treg cells in CD4^+^ T cells (from 10.6 to 2.25%; *p* < 0.001; Additional file 1: Fig. S8C and F). This suggested that combining the MMP2/9 inhibitor SB-3CT with anti-CTLA-4 can promote active lymphocytes and reduce suppressed immune cells and PD-L1 to enhance therapeutic efficacy.

### SB-3CT improved therapeutic efficacy of ICB therapy in lung metastasis models of melanoma

**Fig. 7:**
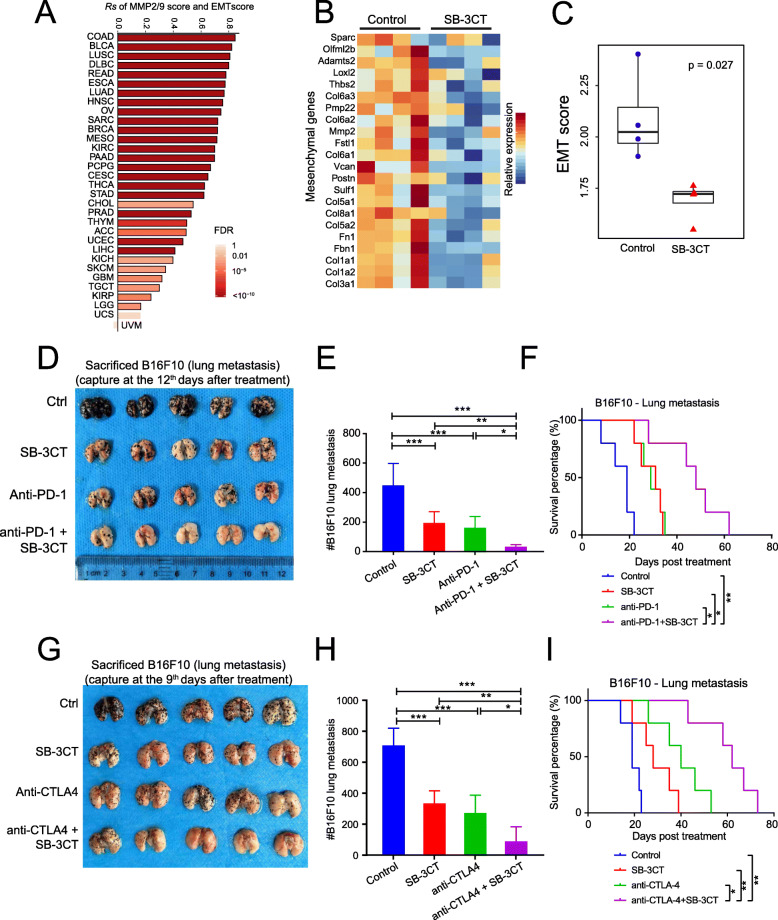
SB-3CT significantly reduces melanoma lung metastasis in combination with checkpoint inhibition. **a** Spearman’s correlation between MMP2/9 score and epithelial-to-mesenchymal transition (EMT) score. **b** Heatmap shows the relative expression of mesenchymal genes differentially expressed between B16F10 tumor with vehicle (*n* = 4) and SB-3CT treatment (*n* = 4). **c** EMT score between SB-3CT treatment and vehicle group. *p* value is determined by two-sided Student’s *t* test. **d** Representative images and **e** quantification of lung metastasis in wild-type C57/BL6 mice with B16F10 cells intravenously treated with isotype, SB-3CT, anti-PD-1, or combination strategy. **f** Kaplan–Meier survival curve for mice over time. **g** Representative images and **h** quantification of lung metastasis in wild-type C57/BL6 mice with B16F10 cells intravenously treated with isotype, SB-3CT, anti-PD-1, or combination strategy. **i** Kaplan–Meier survival curve for mice over time. Sample size per group in one experiment is 5. Error bars represent s.d. of individual mice per group in one experiment. NS, *p* > 0.05, *p < 0.05, **p < 0.01, and ***p < 0.001, as determined by **b**, **c** two-way ANOVA, **f**, **i** two-sided log-rank test, and **e**, **h** one-way ANOVA and Dunnett’s multiple comparison test

Metastasis is the process in which tumor cells migrate from the primary site to distant organs. Despite recent advances in treatment, it remains the leading cause of cancer-related death [40]. Our analysis demonstrated a strong correlation between the group4 score and the EMT pathway across 31 cancer types (median *Rs* = 0.66; Fig. 7a), which is consistent with previous observations [10, 11]. Indeed, B16F10 tumors administered SB-3CT treatment showed downregulation of multiple mesenchymal genes (Fig. 7b), including Col3a1 (fold change [FC] = 3.35, *p* = 0.019), Col1a2 (FC = 3.2, *p* = 0.022), and Fbn1 (FC = 2.48, *p* = 0.037). The EMT score was significantly decreased after SB-3CT treatment (Fig. 7c). We investigated the efficacy of SB-3CT and anti-PD-1 in combination or alone as treatment of B16-F10 melanoma with lung metastasis (Additional file 1: Fig. S9A). The combination of SB-3CT and anti-PD-1 to treat mice with B16F10 tumors significantly reduced lung metastasis (Fig. 7d, e) and substantially extended the survival time of the tumor-bearing mice (48 days vs 19 days; *p* < 0.01; Fig. 7f). In addition, the combination of SB-3CT and anti-CTLA-4 to treat this mouse model tumor (Additional file 1: Fig. S9B) showed similar results that substantially inhibited lung metastasis (Fig. 7g, h) and extended the survival time of the mice with tumor metastasis to the lungs (62 days vs 19 days; *p* < 0.01; Fig. 7i). As described in the extensive literature, intravenous injection and counting of pulmonary modules is a classical model of the later stages of metastasis or colonization by melanoma [41–46]. Nevertheless, it will also be interesting to know if the lungs from the subcutaneously injected mice showed a similar pattern. Taken together, our results suggested the clinical utility of SB-3CT to improve the therapeutic efficacy of ICB therapy in metastatic tumors (Fig. 7).

## Discussion

Therapies for treating cancer by utilizing the human immune system have shown substantial clinical benefits. However, the overall response rate to PD-1/PD-L1 blockers has been relatively low, so it is necessary to further improve ICB therapy by combining it with other treatment strategies. MMPs are the most prominent family of proteinases associated with tumorigenesis. There are increasing studies that reported the associations between MMP family and cancer immunotherapy. For example, Juric et al. reported that anti-MMP-9 treatment can increase the expression of CXCL10 and other T cell-related stimulatory factors, including IL-12p70 and IL18 [47]. However, the association of MMPs and immune checkpoint blockade therapy, particularly, whether it is involved in the regulation of PD-L1, remains unclear. Herein, we systematically explored the dysregulation of MMPs in human cancers and demonstrated that MMP2/9 highly correlates with TILs. MMP2/9 is also associated with T cell exhaustion and inhibitory immune checkpoints. We further demonstrated that SB-3CT, an MMP2/9 inhibitor, significantly enhances T cell–mediated cytotoxicity. Our results showed that MMP2/9-specific inhibitor SB-3CT significantly decreased PD-L1 mRNA and protein levels in melanoma cell lines and significantly reduced the PD-L1 protein level in PD-L1 overexpression cell line. Based on our results and previous literatures, we speculate that MMP inhibition may decrease PD-L1 expression by inactivating TGF-β. It was reported that MMPs (MT1-MMP, MMP2, and MMP9) can activate TGF-β via proteolytic cleavage of latent transforming growth factor-β binding protein (LTBP) and the latency-associated peptide (LAP) [48–50]. Additionally, Tumor growth factor beta (TGF-β) can significantly elevate the PD-L1 expression by inducing EMT [51, 52]. Therefore, we hypothesize that MMP inhibition may inactivate TGF-β, leading to the PD-L1 downregulation. Our previous study and many recent studies have shown that the tumor PD-L1 expression level affects tumor immunity by control of cytotoxic T cell activity [53–58]. Therefore, our results suggested that tumor PD-L1 may contribute to SB-3CT-mediated anti-tumor effect. It is necessary to perform experiments in in vitro and in vivo assays with deficient PD-L1 to test if SB-3CT requires PD-L1 for its efficacy.

According to the ClinicalTrials.gov and previous literatures, several MMP inhibitors were synthesized and trialed [18–20, 59]. Unfortunately, several clinical trials of MMP inhibitors are failed at different phases, mainly due to the non-specificity of the drug and the complicated background for specific effects of MMPs. The development of MMP inhibitors is largely paused due to these failures. New clinical trials of MMP inhibitors have been started in cancers to improve the therapeutic efficacy through combination treatment with other therapies or compounds. For example, MMP9 neutralizing antibody (andecaliximab) and anti-PD1 (nivolumab) was combined to treat gastric and esophageal cancers in the phase II clinical trial NCT02864381 [60], suggesting the potential re-application of MMP inhibitors in cancer therapy. Our in vivo studies showed that SB-3CT, an MMP2/9 inhibitor, could improve the efficacy of anti-PD-1 and anti-CTLA4 treatment in mouse models with melanoma and lung cancer, as well as metastatic melanoma in the lung. Through our computational analysis and in vitro assays, we revealed a potential mechanism in that MMP2/9 modulates the expression of PD-L1 in tumor cells. As a small molecule, SB-3CT is much cheaper to use than anti-PD-1 or anti-CTLA4 antibodies [61]. More importantly, SB-3CT targets MMP2/9 in the tumor microenvironment rather target immune response directly, which is how antibodies function, and it may cause less toxicity or immune-related adverse events, thus making SB-3CT a potential reagent for future immunotherapy [62–64]. Further studies are necessary to examine the potential toxicity of SB-3CT or its associated immune-related adverse events.

Our work provides a novel paradigm for reducing the workload for identifying potential reagents for immunotherapy through integrative analysis as well as in vitro assays. We subdivided MMPs based on the structure of functional domains and their substrate specificities as the previous study suggested, including signal peptide domain, propeptide domain, catalytic domain, and haemopexin-like domain [10]. This is a widely accepted classification, which is used in many other studies [10, 65–67]. It is possible to subdivide MMPs based on different criteria, which may lead to the discoveries of other MMP inhibitors that may play significant roles in clinical practice.

## Conclusions

MMP2/9 is significantly dysregulated in human cancers and is associated with poor prognosis and detrimental immune features. The inhibition of MMP2/9 by SB-3CT significantly reduced the tumor burden and improved survival time via the promotion of anti-tumor immunity, potentially through reducing PD-L1 expression. SB-3CT treatment enhanced the therapeutic efficacy of PD-1 or CTLA-4 blockade in the treatment of both primary and metastatic tumors, thus providing a novel therapeutic strategy for SB-3CT and ICB therapy to enhance immunotherapy efficacy.

## Supplementary information

**Additional file 1: Fig. S1.** MMP2/9 associated with poor prognosis and cancer hallmarks. **Fig. S2.** Schematic of experimental tumor-bearing model for the combination treatment of SB-3CT and PD-1 blockade. **Fig. S3.** Expression of TILs in tumors from B16F10 xenograft mouse model with SB-3CT and PD-1 blockade. **Fig. S4.** Association of MMP2/9 score and PD-L1. **Fig. S5.** PD-L1 Expression through MMP2/9, SB-3CT with limited activity in the context of KD of MMP2 and MMP9. **Fig. S6.** Combination treatment of SB-3CT and CTLA-4 blockade in tumor-bearing model. **Fig. S7.** Expression of TILs in tumors from B16F10 xenograft mouse model with SB-3CT and CTLA-4 blockade. **Fig. S8**. Infiltration of functional immune cells in tumors from B16F10 xenograft mouse model with SB-3CT and CTLA-4 blockade. **Fig. S9.** Effects of SB-3CT in mouse model of B16F10 tumor with lung metastasis.

## Data Availability

The RNA-seq data for SB-3CT treatment have been submitted to the NCBI Gene Expression Omnibus with accession number GSE141585 [68]. All public datasets analyzed in this study are listed in the Methods “Data collection and processing” section.
