# Supplementary material for: Small-molecule MMP2/MMP9 inhibitor SB-3CT modulates tumor immune surveillance by regulating PD-L1
Source: Genome Med. 2020 Sep 28;12:83. doi: 10.1186/s13073-020-00780-z (PMC7523356; doi:10.1186/s13073-020-00780-z)
Supplement: Supplementary file 1 — Additional file 1: Fig. S1. MMP2/9 associated with poor prognosis and cancer hallmarks. Fig. S2. Schematic of experimental tumor-bearing model for the combination treatment of SB-3CT and PD-1 blockade. Fig. S3. Expression of TILs in tumors from B16F10 xenograft mouse model with SB-3CT and PD-1 blockade. Fig. S4. Association of MMP2/9 score and PD-L1. Fig. S5. PD-L1 Expression through MMP2/9, SB-3CT with limited activity in the context of KD of MMP2 and MMP9. Fig. S6. Combination treatment of SB-3CT and CTLA-4 blockade in tumor-bearing model. Fig. S7. Expression of TILs in tumors from B16F10 xenograft mouse model with SB-3CT and CTLA-4 blockade. Fig. S8. Infiltration of functional immune cells in tumors from B16F10 xenograft mouse model with SB-3CT and CTLA-4 blockade. Fig. S9. Effects of SB-3CT in mouse model of B16F10 tumor with lung metastasis. [file 13073_2020_780_MOESM1_ESM.pdf]

Small-molecule MMP2/MMP9 inhibitor SB-3CT modulates tumor immune surveillance by regulating PD-L1

Youqiong Ye[2,3,#,*], Xinwei Kuang[1,4,5,#], Zuozhong Xie[1,4,5,#], Long Liang[1,6], Zhao Zhang[3], Yongchang Zhang[7], Fangyu Ma[8], Qian Gao[1,4,5], Ruimin Chang[1,4,5], Shuang Zhao[1,4,5], Juan Su[1,4,5], Hui Li[6], Jingbo Peng[9], Huifang Chen[2], Minzhu Yin[1,4,5], Cong Peng[1,4,5], Nong Yang[7], Jing Liu[6], Hong Liu[1,4,5,10*], Leng Han[3*], Xiang Chen[1,4,5*]

[1]Department of Dermatology, Xiangya Hospital, Central South University, Changsha, Hunan 410013, China.

[2]Shanghai Institute of Immunology, Department of Immunology and Microbiology, State Key Laboratory of Oncogenes and Related Genes, Shanghai Jiao Tong University School of Medicine, Shanghai, 200025, China.

[3]Department of Biochemistry and Molecular Biology, The University of Texas Health Science Center at Houston McGovern Medical School, Houston, TX 77030, USA

[4]Hunan Key Laboratory of Skin Cancer and Psoriasis, Changsha, Hunan 410013, China.

[5]Hunan Engineering Research Center of Skin Health and Disease, Changsha, Hunan 410013, China.

[6]Medical Genetics & School of Life Sciences, Central South University, Changsha, Hunan 410013, China.

[7]Department of medical oncology, lung cancer and gastrointestinal unit, Hunan cancer hospital/The Affiliated Cancer Hospital of Xiangya School of Medicine, Central South University, Changsha, Hunan 410013, China.

[8]Department of Health Management Center, Xiangya Hospital, Central South University, Changsha, Hunan 410013, China

[9]Department of Clinical Pharmacology, Xiangya Hospital, Central South University, Hunan 410013, China

[10]Research Center of Molecular Metabolomics, Xiangya Hospital, Central South University, Changsha, Hunan, China.

[#]These authors contributed equally to this work.

* Correspondence: chenxiangck@126.com (X. C.), leng.han@uth.tmc.edu (L.H.), hongliu1014@csu.edu.cn (H. L.), youqiong.ye@shsmu.edu.cn (Y.Y)

Figure S1.

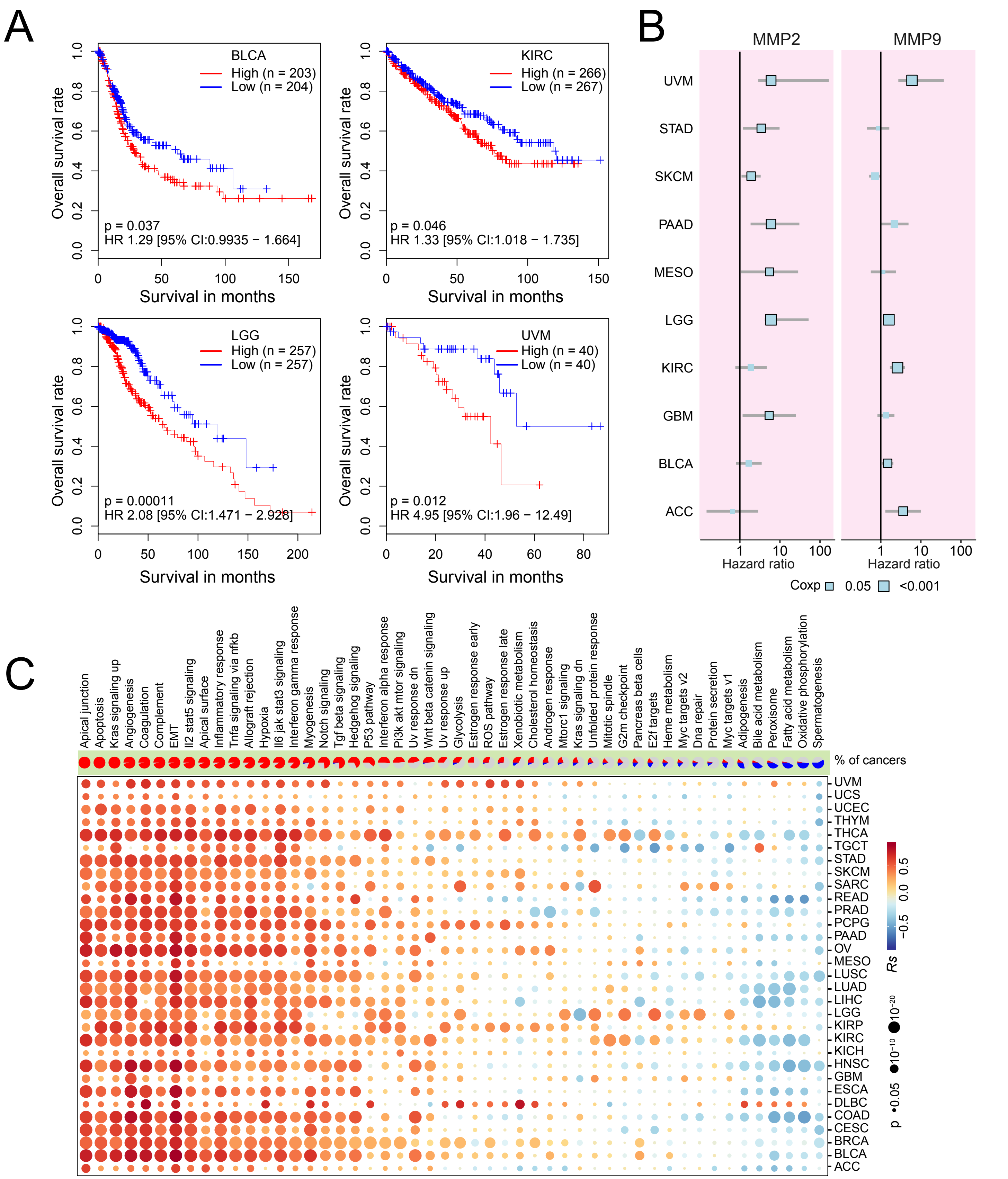

Fig. S1. MMP2/9 associated with poor prognosis and cancer hallmarks. (A) Kaplan–Meier curves show overall survival time stratified by median expression levels of top genes of group4 score (two-sided log-rank test P < 0.05 considered statistically significant). (B) MMP2/9 associated with overall survival analysis using a Cox proportional hazards model; x-axis indicates higher expression of MMP2/9 with worse (hazard ratio [HR] > 1) or better (HR < 1) overall survival. Black border outside the point indicates p value < 0.05. Black vertical lines indicate 95% confidence interval (CI). (C) Spearman's correlation between group4 score and MSigDB hallmark pathways (x-axis) across 33 cancer types (y-axis). Size indicates FDR; color indicates Spearman's correlation coefficient (Rs). Pie charts in upper panel represent the percentage of cancer types with positive (red; Rs > 0.2, FDR < 0.05), negative (blue; Rs < -0.2, FDR < 0.05) or non-significant (gray) correlation.

Figure S2.

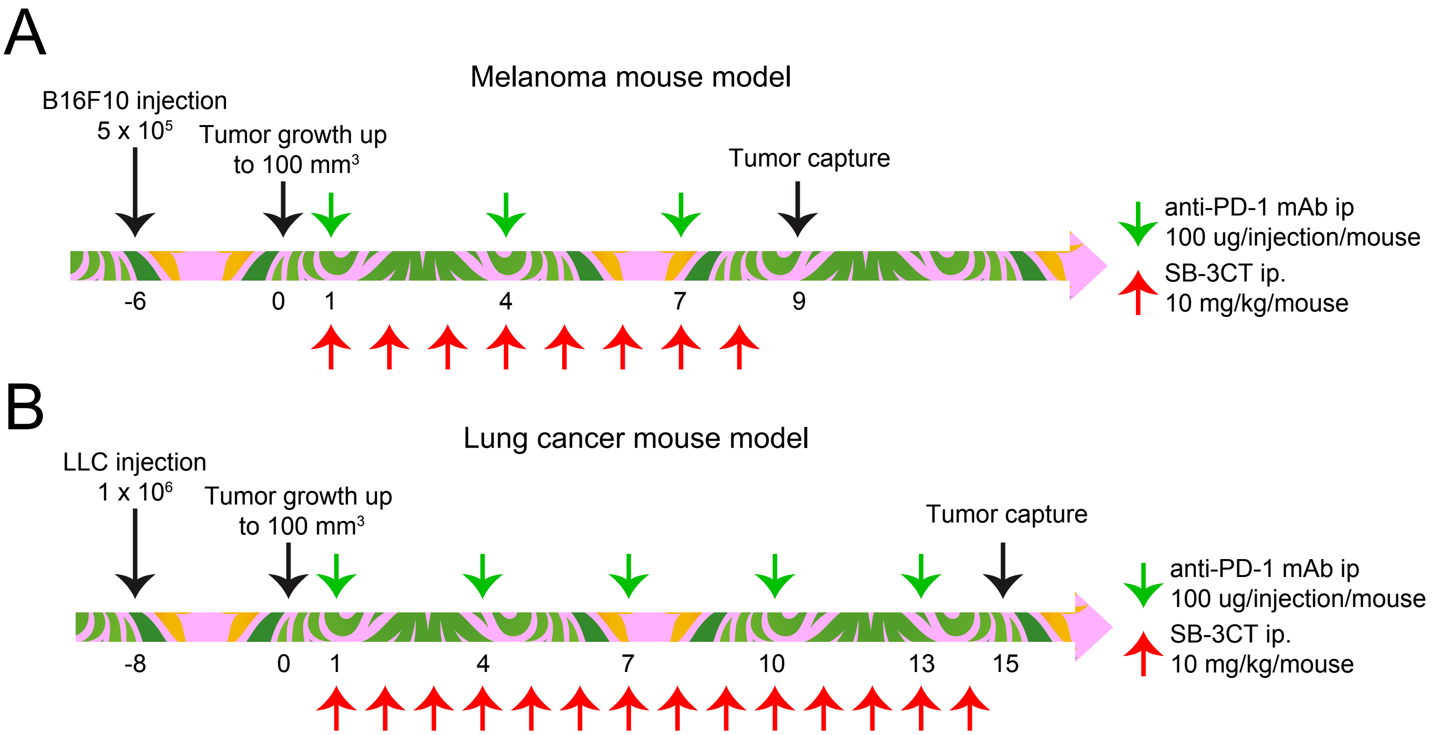

Fig. S2. Schematic of experimental tumor-bearing model for the combination treatment of SB-3CT and PD-1 blockade. Treatment plan schematic for mice bearing subcutaneous (A) B16f10 cells or (B) LLC cells.

Figure S3.

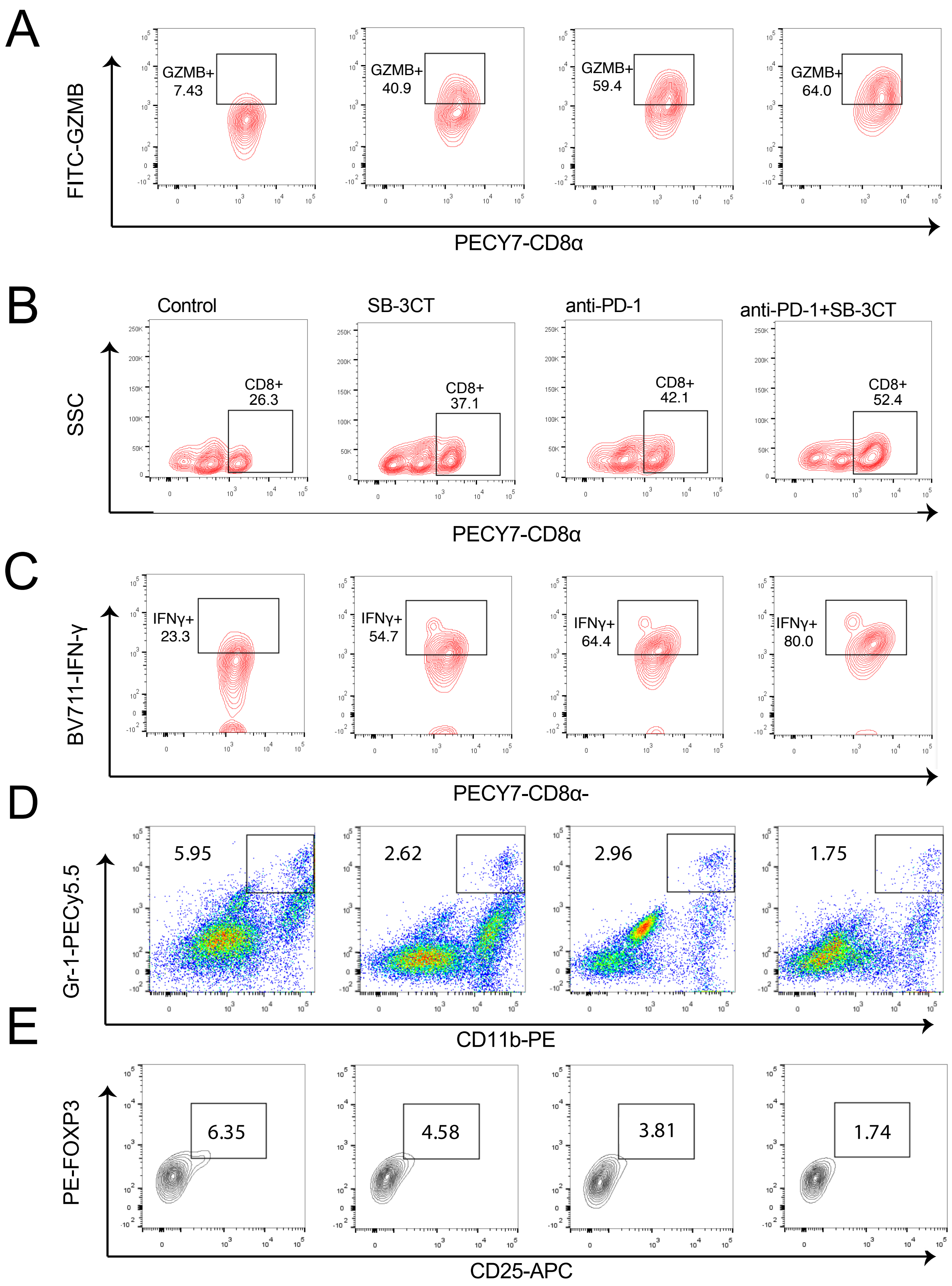

Fig. S3. Expression of TILs in tumors from B16F10 xenograft mouse model with SB-3CT and PD-1 blockade. Fluorescence-activated cell sorting (FACS) of (A) CD8+ in CD3+ T cells, (B) CD8+ IFNγ+ in CD8+ T cells, (C) CD8+ GZMB+ in CD8+ T cells, (D) Gr-1+ CD11b+ MDSCs in CD45+ cells, and (E) CD25+ FOXP3+ Treg in CD4+ cells, in B16F10 tumor-bearing wild-type C57/BL6 mice treated with isotype, SB-3CT, anti-PD-1 or combination strategy.

Figure S4.

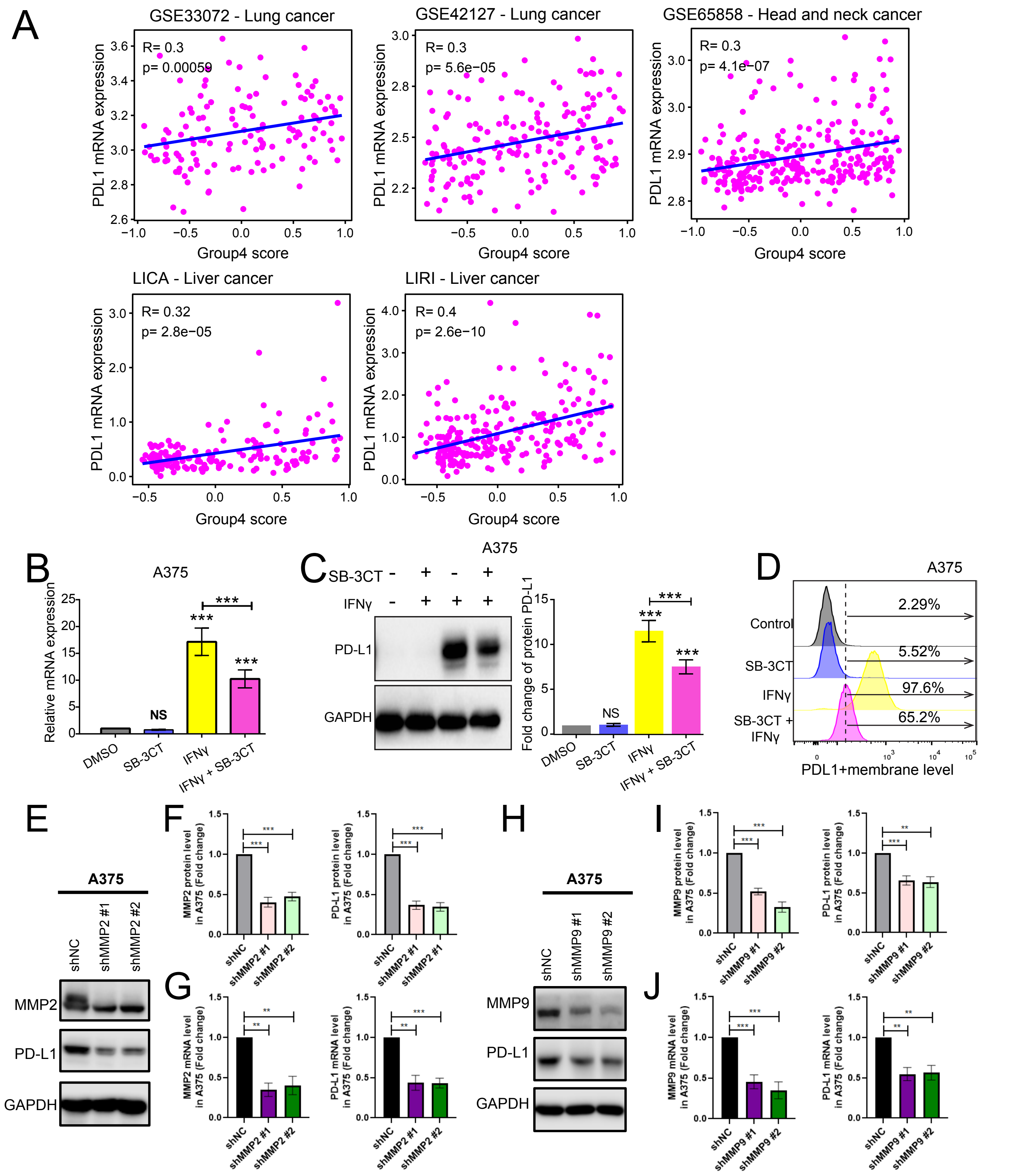

Fig. S4. Association of MMP2/9 score and PD-L1. (A) Correlation of mRNA expression of PD-L1 across independent datasets with multiple cancer types, including lung cancer (GSE33072: n = 131, GSE42127: n = 176), head and neck cancer (GSE65858: n = 270) and liver cancer (LICA: n = 160, LIRI: n = 232). (B) RT-PCR of relative PD-L1 mRNA expression, (C) western blot (left panel: representative images, right panel: quantification) of PD-L1 protein levels and (D) flow cytometry of PD-L1+ membrane level in A375 melanoma cancer line treated with DMSO, SB-3CT (25μM), IFNγ (200ng/mL), and IFN γ/SB-3CT in combination for 24 h. (E-J) PD-L1 expression of A375 melanoma cell line transfected with shMMP2 (E-G), shMMP9 (H-J) or the scrambled negative control shRNA (shNC). Western blot (E, H), quantification of MMP2, MMP9, and PD-L1 protein expression (F, I), RT-PCR analysis of MMP2, MMP9, and PD-L1 mRNA expression (G, J). All experiments were repeated three times independently. Results are displayed as mean ± s.d.. P values are determined by one-way ANOVA and Dunnett's multiple comparison test (NS, $p > 0.05$, *$p < 0.05$, **$p < 0.01$, and ***$p < 0.001$).

Figure S5.

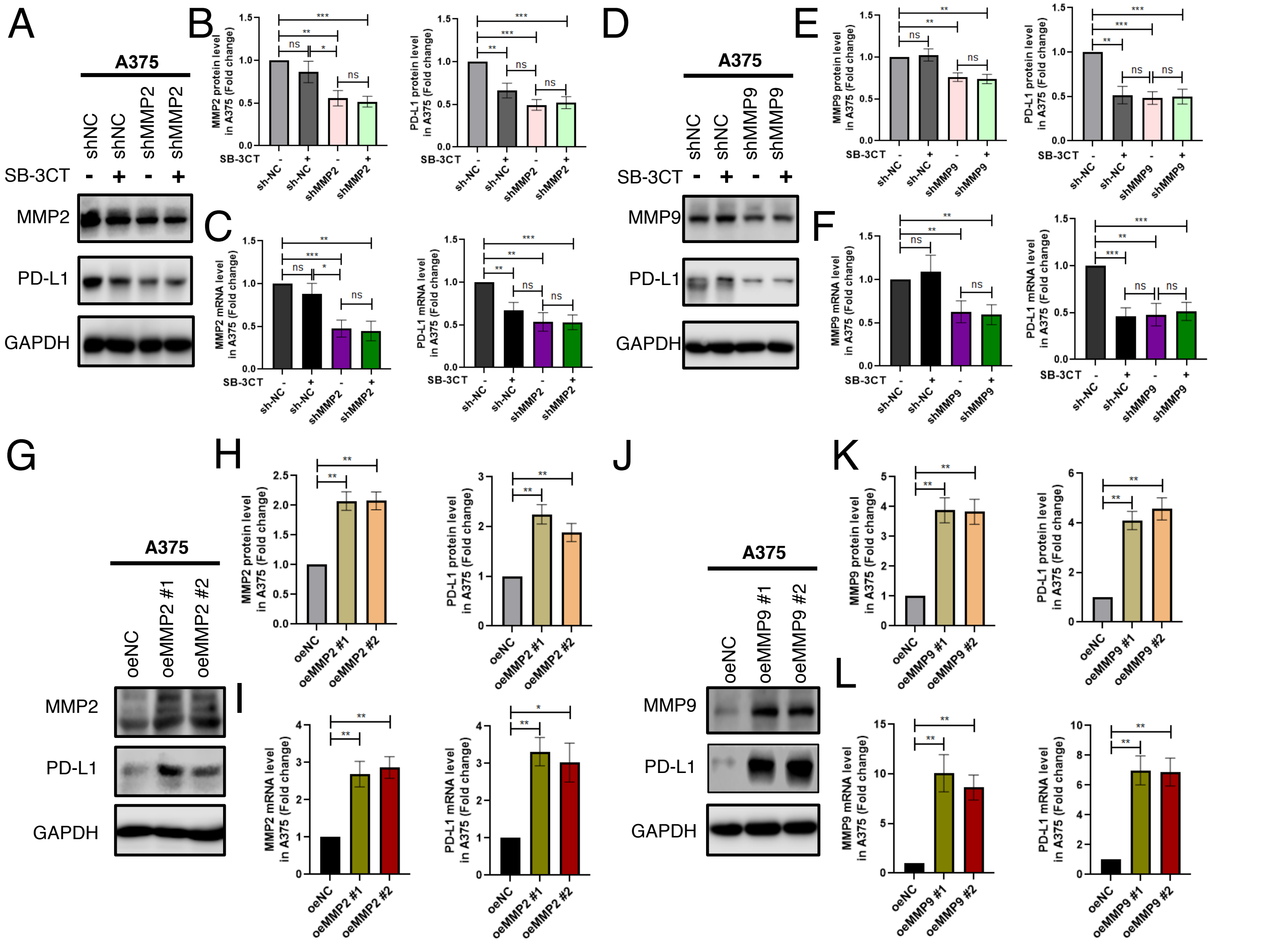

Fig. S5. PD-L1 Expression through MMP2/9, SB-3CT with limited activity in the context of KD of MMP2 and MMP9. (A-F) PD-L1 expression of shMMP2 (A-C), shMMP9 (D-F) A375 melanoma cell line treated with SB-3CT. Western blot (A,D), quantification, of MMP2, MMP9, and PD-L1 protein (B, E) and mRNA expression (C, F). (G-L) Analysis of PD-L1 expression of A375 melanoma cell line with overexpression (oe) MMP2 (G-I), oeMMP9 (K-L). (G, J) Western blot, (H, K) quantification, and RT-PCR analysis (I, L) of MMP2, MMP9, and PD-L1 protein or mRNA expression. Results are mean ± s.d. *p < 0.05, **p < 0.01, and ***p < 0.001, as determined by one-way ANOVA and Dunnett's multiple comparison test.

Figure S6.

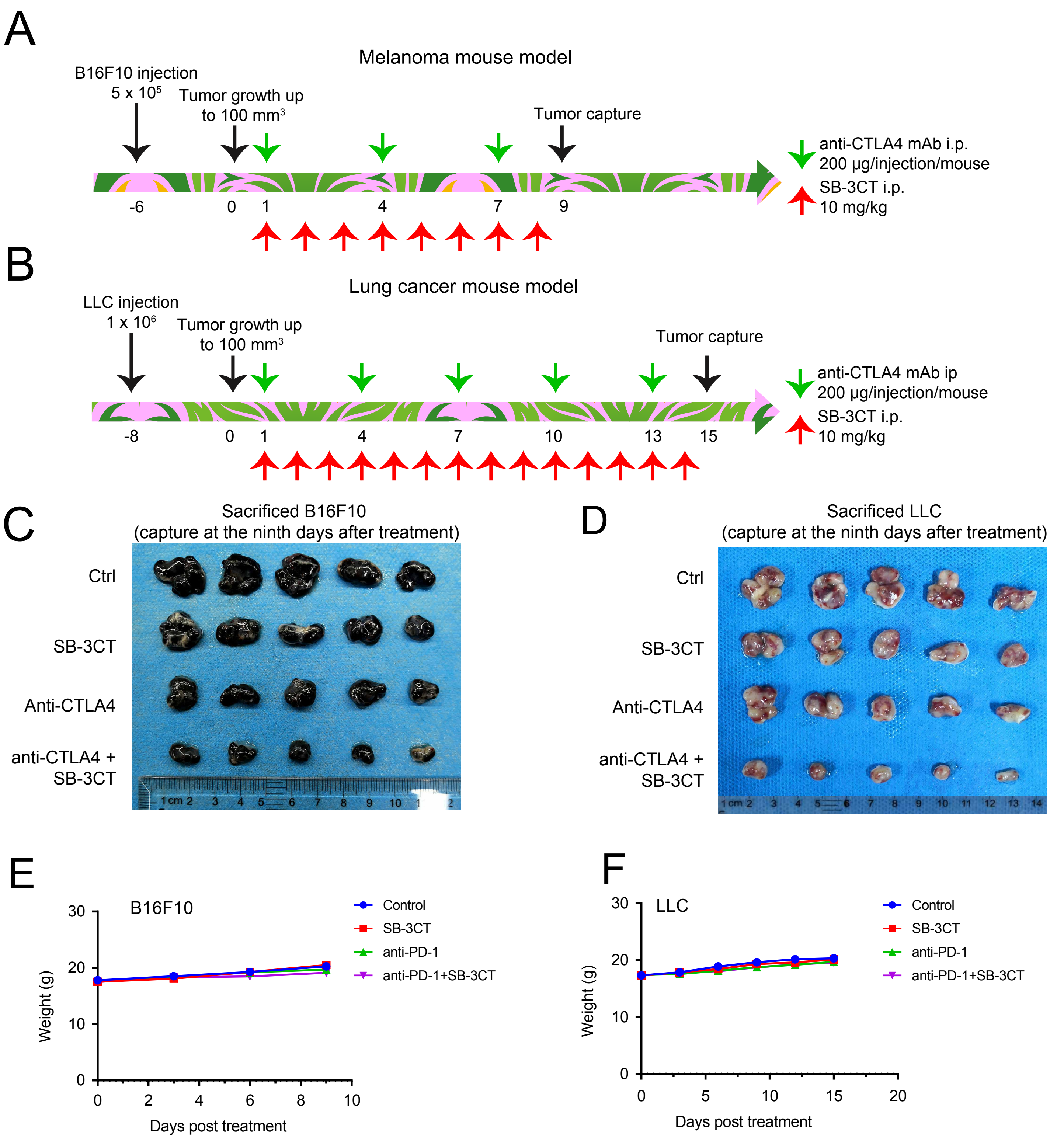

Fig. S6. Combination treatment of SB-3CT and CTLA-4 blockade in tumor-bearing model. Schematic of the treatment plan for mice bearing subcutaneous (A) B16f10 cells or (B) LLC1 cells. Images of (C) B16F10 tumor and (D) LLC tumor collected from mice after different combination treatment strategies (control: isotype; PD1 inhibition: anti-PD1; MMP2/9 inhibition: SB-3CT; combination treatment: anti-CTLA-4 + SB-3CT). (E-F) Body weight of (G) B16F10 and (H) LLC tumor-bearing C57/BL6 mice treated with isotype, SB-3CT, anti-CTLA-4 or combination strategy.

Figure S7.

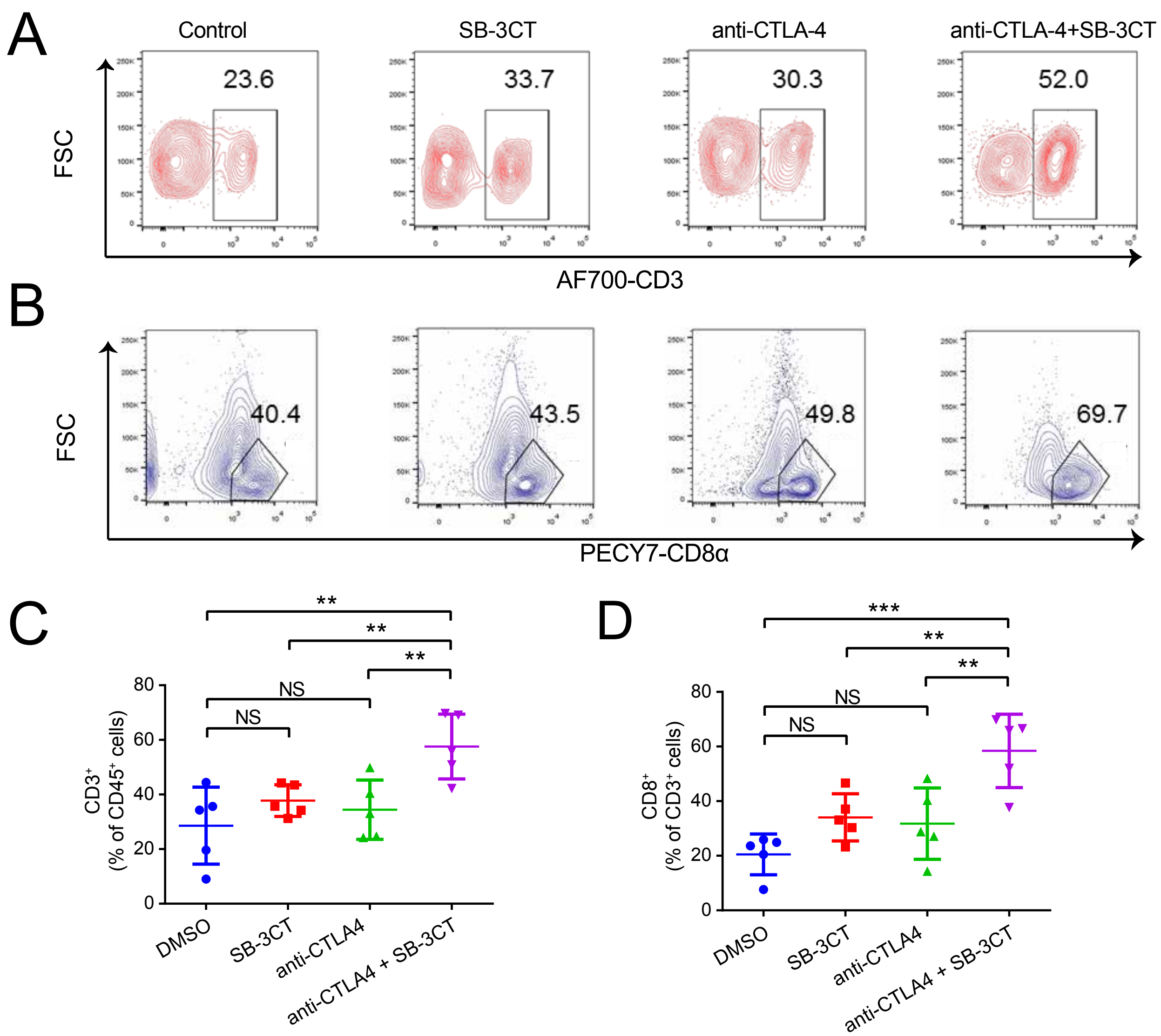

Fig. S7. Expression of TILs in tumors from B16F10 xenograft mouse model with SB-3CT and CTLA-4 blockade. (A and B) Fluorescence-activated cell sorting (FACS) and (C and D) quantification of immune infiltration, including CD3+ in CD45 cells and CD8+ in CD3+ T cells.

Figure S8.

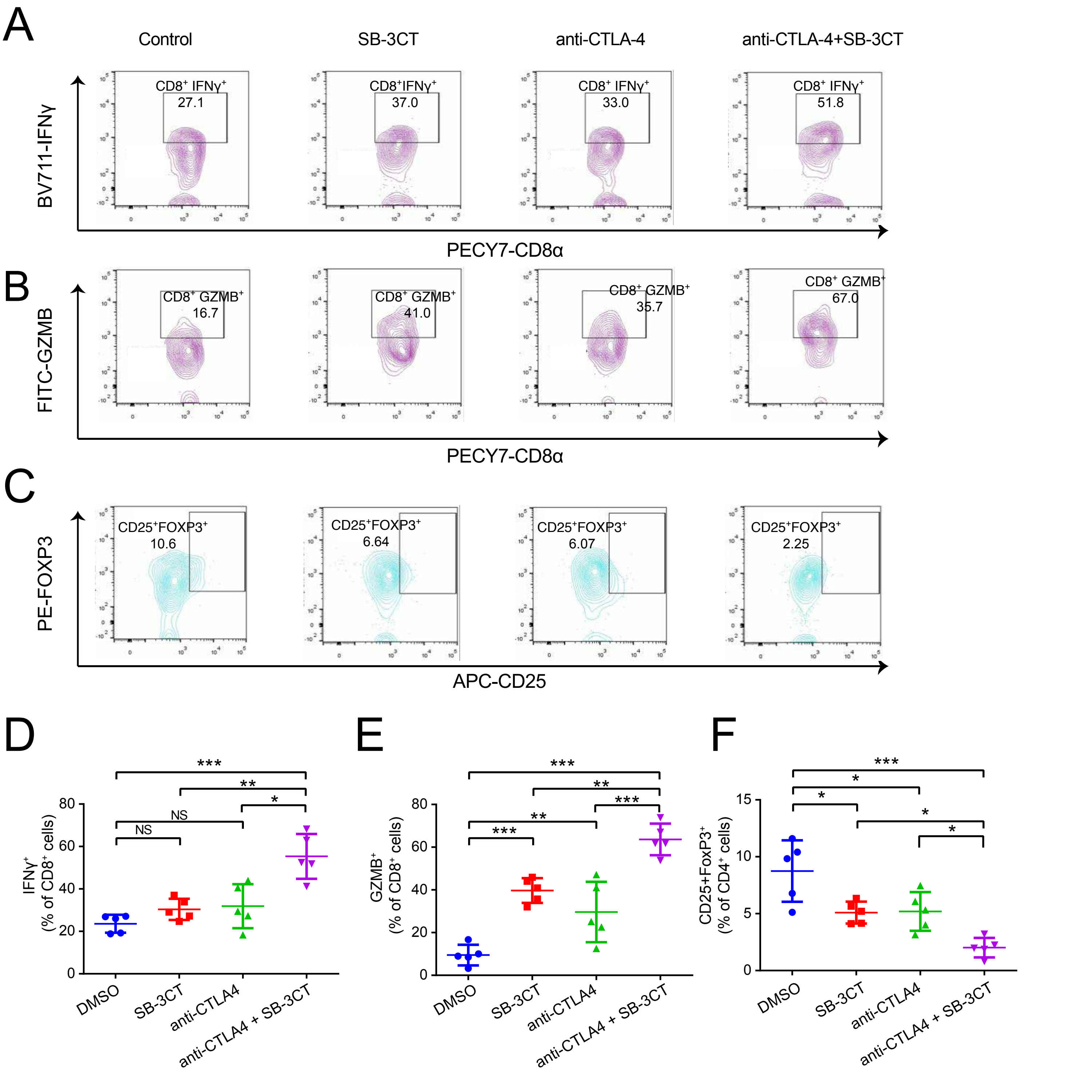

Fig. S8. Infiltration of functional immune cells in tumors from B16F10 xenograft mouse model with SB-3CT and CTLA-4 blockade. (A-C) Fluorescence-activated cell sorting (FACS) and (D-F) quantification of immune infiltration, including (A and D) CD8+ IFNγ+ in CD8+ T cells, (B and E) CD8+ GZMB+ in CD8+ T cells, and (C and F) CD25+ FOXP3+ Treg in CD4+ cells.

Figure S9.

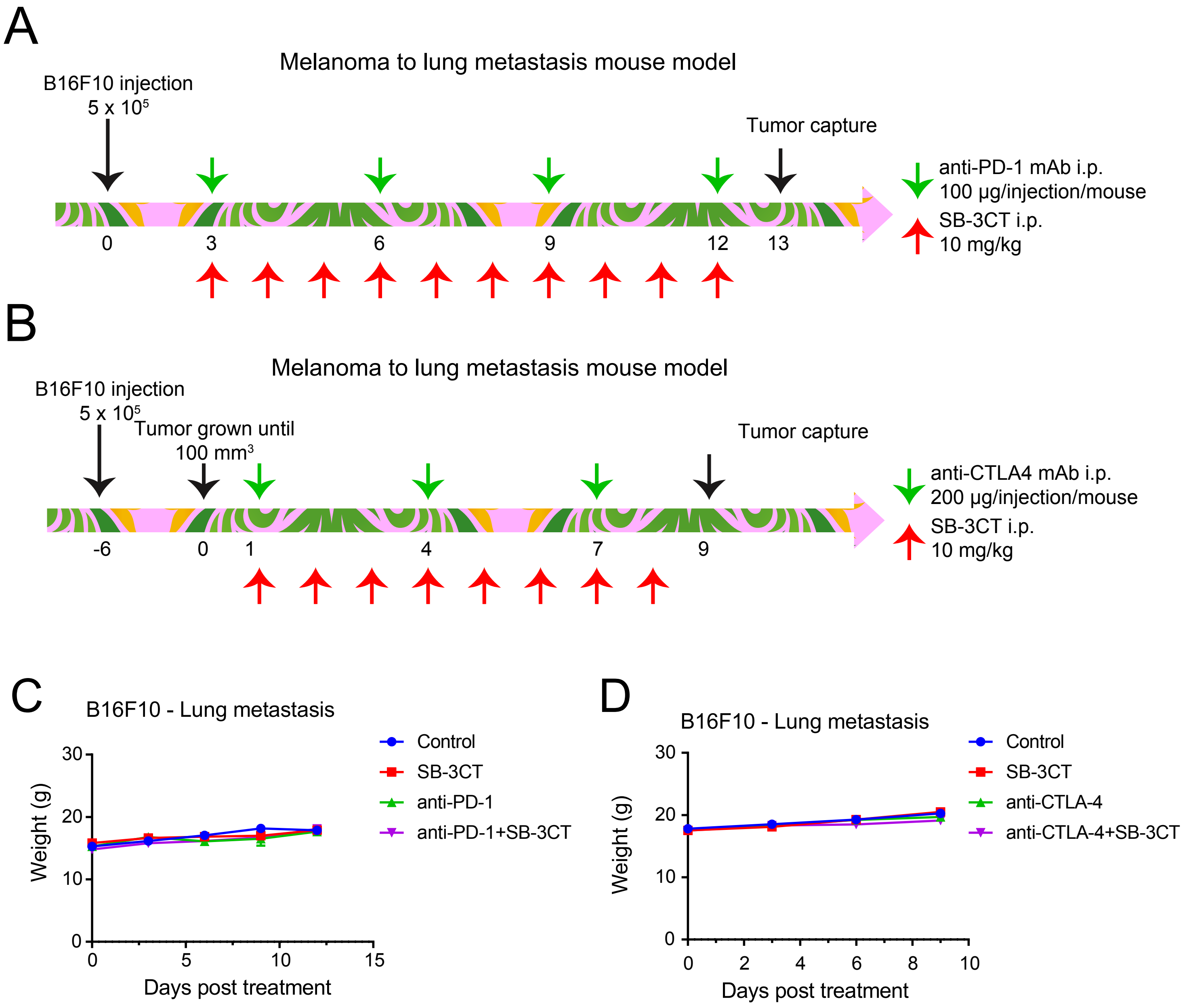

Fig. S9. Effects of SB-3CT in mouse model of B16F10 tumor with lung metastasis. (A-B) Treatment plan schematic for mice bearing lung metastasis of B16F10 tumor cells with SB-3CT treatment combined with (A) anti-PD-1 or (B) anti-CTLA-4. (C-D) Body weight of lung metastasis with B16F10 cells of tumor-bearing C57/BL6 mice treated with SB-3CT in combination with (C) anti-PD-1 or (D) anti-CTLA-4. Results are displayed as mean ± s.d..
